# Estimating Impacts of Climate Change Policy on Land Use: An Agent-Based Modelling Approach

**DOI:** 10.1371/journal.pone.0127317

**Published:** 2015-05-21

**Authors:** Fraser J. Morgan, Adam J. Daigneault

**Affiliations:** Landcare Research, Auckland, New Zealand; University of Maryland at College Park, UNITED STATES

## Abstract

Agriculture is important to New Zealand’s economy. Like other primary producers, New Zealand strives to increase agricultural output while maintaining environmental integrity. Utilising modelling to explore the economic, environmental and land use impacts of policy is critical to understand the likely effects on the sector. Key deficiencies within existing land use and land cover change models are the lack of heterogeneity in farmers and their behaviour, the role that social networks play in information transfer, and the abstraction of the global and regional economic aspects within local-scale approaches. To resolve these issues we developed the Agent-based Rural Land Use New Zealand model. The model utilises a partial equilibrium economic model and an agent-based decision-making framework to explore how the cumulative effects of individual farmer’s decisions affect farm conversion and the resulting land use at a catchment scale. The model is intended to assist in the development of policy to shape agricultural land use intensification in New Zealand. We illustrate the model, by modelling the impact of a greenhouse gas price on farm-level land use, net revenue, and environmental indicators such as nutrient losses and soil erosion for key enterprises in the Hurunui and Waiau catchments of North Canterbury in New Zealand. Key results from the model show that farm net revenue is estimated to increase over time regardless of the greenhouse gas price. Net greenhouse gas emissions are estimated to decline over time, even under a no GHG price baseline, due to an expansion of forestry on low productivity land. Higher GHG prices provide a greater net reduction of emissions. While social and geographic network effects have minimal impact on net revenue and environmental outputs for the catchment, they do have an effect on the spatial arrangement of land use and in particular the clustering of enterprises.

## Introduction

Agriculture and Forestry are a significant part of New Zealand’s economy, generating 70% of its export merchandise earnings and about 12% of its GDP [1]. As the sector strives to maintain or enhance the level of output, it faces a range of environmental challenges that need to be accounted for or mitigated. The use of additional inputs, including fertilizer, irrigation, and supplemental feeds, contributes to a significantly increased level of agricultural productivity in most regions of the country. The intensifying agricultural inputs result in increased nutrient leaching levels and sediment runoff to lakes, rivers and streams, putting additional strain on the country’s freshwater resources. Approximately 46% of New Zealand’s total greenhouse gas (GHG) emissions occur in the agricultural sector, which is high compared with other developed countries in the world [2].

In 2008, New Zealand implemented an Emissions Trading Scheme (ETS) to reduce its GHG emissions. The policy is unique as it currently covers all major sectors of the economy, including forestry, and discussions are on-going about the most appropriate way to bring the agricultural sector into the ETS to help meet national emissions targets without placing a large burden on its stakeholders. As of 2014, agriculture’s primary points of obligation will be the meat, wool and dairy processors. Processing companies are likely to adopt measures to pass that obligation upstream to the farming community, so farmers can expect to face a GHG price, albeit indirectly through the processors. In addition, farmers are also subject to a GHG price through, for example, higher fuel prices as fuel suppliers pass on the cost of their climate obligations to consumers.

Understanding the spatial effects of the ETS on land use alongside the economic and productivity outputs is critical in understanding the wider implications of a GHG price. Modelling is used to understand the implications of the ETS because the social, economic and geographic factors that determine the choice and impact of land use are complex [3–5]. Most of the approaches to model economic and land-use impacts of environmental policy are focused on a particular impact or limited by its model structure.

Land use and land cover change (LULCC) models are a well-developed approach to modelling and understanding the processes which shape the environment around us [6–8]. LULCC models have developed substantially alongside our understanding of wider economic and social systems. As is the case in most modelling approaches, early implementation focused on mathematical and equilibrium methodologies through models that concentrated on the economic aspects of LUCC [9–13]. These approaches are still common and work on expecting LULCC actions to follow the rules of rational utility theory in which a land-use pattern can be explained in terms of actors who maximise their utility. These models capture the trend of LULCC, but are often unable to explain or examine the processes that caused it [14].

LULCC is regarded as a complex adaptive system that can be explained and simulated more effectively through the use of computational tools like Agent-Based Modelling (ABM) [14]. Within LULCC models, ABMs are used because they are particularly well suited for representing complex spatial interactions under heterogeneous conditions and for modelling decentralised, autonomous decision making [14]. In addition the approach introduces the concepts of space, distance, and time, while allowing agent behaviours and types to be implemented within a model of land-use transition.

From a rural LULCC perspective, capturing the social and economic behaviour of farmers to enable an agent- based approach is a problematic task [15]. Burton [16] outlines the numerous issues that could be accounted for when examining farmers and their behaviours. These issues range from cultural embeddedness [17], variation in social networks and the use of these networks for information and technology transfer [18, 19], to the dichotomy between social and economic approaches to farming [20, 21]. Consequently, it is important to capture the heterogeneity of farmers and their behaviour when modelling rural land-use change.

While ABMs are used to investigate a range of policy influences on LULCC from both a geographical [14, 22–27] and economics perspectives [28, 29], the coordination between geographers and economists to conduct these analyses are limited. This has led to a call for modellers from these two disciplines to coordinate their expertise and efforts to build models that can provide a better representation of the real world [28, 30]. While there are a range of rural LULCC models developed for New Zealand [31–35], the use of ABMs is limited with only Kaye-Blake et al. [36], who used an agricultural ABM entitled RF-MAS (Rural Futures Multi-Agent Simulation) to project land use change in NZ’s Southland region. Developed independently from the ARLUNZ model, the RF-MAS model focused on the agricultural production at a regional scale, but is not spatially explicit.

Utilising the ABM approach, this paper presents a spatially explicit agent-based economic model titled “Agent-based Rural Land Use New Zealand” that is capable of analysing the impact of a variety of policies on farm level-land use, farm net revenue, and environmental indicators such as GHG emissions, nutrient loadings and soil erosion. The model can also assess the resulting land-use effects caused by changes in farming demographics, social networks, and decision making. Using this model we explore the spatial effects on land use, through changes in a GHG emissions price on the forest and agricultural sector, and the effects of information diffusion through farmer social networks.

## Methods

The Agent-based Rural Land Use New Zealand model (hereafter ARLUNZ) was designed to examine and resolve complex environmental issues within the rural environment, provide information about how farmers will adapt (both economically and socially) to global change, and reduce vulnerability to resource scarcity. Specifically, ARLUNZ implements a spatially and behaviourally heterogeneous population of farmers that operate in line with a real world population.

ARLUNZ is written in Version 5.0.5 of NetLogo [37] using the GIS, String, and Shell extensions. Python 2.7 is used to facilitate a loose coupling [38] between ARLUNZ and a modified version of the New Zealand Forest and Agriculture Regional Model (NZFARM) that provides economic information within ARLUNZ. NZFARM is a comparative-static, non-linear, partial equilibrium mathematical programming model of New Zealand land use operating at the catchment scale [32]. The modified version used within ARLUNZ has been refined to produce an economically optimised result for each farm rather than an optimised landscape for an entire catchment. For more information about NZFARM, Daigneault et al. [32] details its design, parameterisation and validation while Daigneault et al. [39–42] discusses the input data used within model.

## Structure

The model consists of three layers: a landscape on which the agents make decisions; the agents themselves and the associated decision-making framework; and the economic information associated with both the landscape and the agents:

- The landscape holds a range of spatial information about the area being modelled. Initially the cadastral boundaries [43], an initial land use map to define the current land use for each parcel of land [44], and productivity zones (derived using a dataset such as New Zealand’s Land Use Capability (LUC) dataset [45]) are imported.
- Using the cadastral boundaries, an agent is generated at the centroid of each parcel. This ‘farm agent’ does not have any decision-making ability, is immobile, and represents the farm as a whole. The area of the farm is also defined on a 25 hectare raster landscape. The agent also queries the vector datasets for the predominant land use (using the initial land use map) and productivity zone (Plains, Foothills or Hills) within the farm boundary. As the model only simulates the land use conversion between Dairy, Sheep & Beef, and Forestry, any farms with a predominant land use outside of these three enterprises are discarded.
- The farm agent then creates an agent at the same location which represents the farmer. This ‘farmer agent’ encompasses the decision making framework for the model. The agent holds a range of social and economic attributes about the farmer such as the size of their social networks, age, potential revenues, and net revenue from the last step of the model (discussed in more detail under the “Agent Heterogeneity” section below).
- Using the information from the farm and farmer agents, the model determines the optimised use of the farm parcel based on yields, input costs, output prices, and environmental constraints to generate the expected net revenue for the possible land use enterprises available in the model (e.g., Dairy, Sheep & Beef, Pine Plantation, etc.). The land use that generates the highest net revenue for the farm is defined as the land use that each farmer agent assesses for conversion.

Each of the three components (landscape, agent, economic) are integrated through the development of dependencies and feedback loops between each layer, specifically the decision-making framework built around the farmer agent, which takes into account farm, farmers and economic information when making a land use decision.

## Process

Decision making within the model rests entirely with the farmer agent. Once the model is run, farmer (e.g., age, succession, networks) and market (commodity prices) variables are updated. Using the market variables and the spatial and productivity attributes of each farm, the model returns the net revenue-maximising result for the specific farm along with the expected net revenue values for all of the potential enterprises that could be undertaken on that farm.

This result is then compared with the farmer agent’s current enterprise. If these are the same, the net revenue value of the farmer agent is updated to the results provided by economic component of the model. If not, the decision to accept the information is dependent on a stochastic evaluation against the farmer agent’s likelihood of land use conversion. While initially set at 0.2 for all farmer agents, an agent’s likelihood of land use conversion is a function of the information received from the agent’s social and geographical networks, but also their current and the proposed enterprises. To simulate the decision to undertake a land-use conversion, we use a uniform (pseudo) random number generator to generate probabilities for evaluation against the farmer agent's likelihood of land use conversion.

After all farmer agents have assessed their potential for a change in enterprise, the farmer agents who have reached the end of the farming life cycle without finding a successor sell the farms they own. This concludes a time step of the model. The model is run repeatedly until the specified time-step is reached where it halts operation and reports on changes in farmers, farms, economic values and land use.

Each time step in the model represents a 5-year period. We have chosen this interval for two reasons. First, land use change in New Zealand is typically a slow process, with minimal difference when measured on an annual basis [46]. Second, it is aligned with the lengths of different life stages of a farmer as defined by Burton [47].

## Agent heterogeneity

Each farmer agent within the model is heterogeneous in the attributes which shape the agent’s behaviour. It is important to note that in almost all areas where heterogeneity is implemented (apart from Succession), the heterogeneity affects the farmer agent’s likelihood of land use conversion which underpins the primary behaviour being modelled.

### Succession

**Fig 1:**
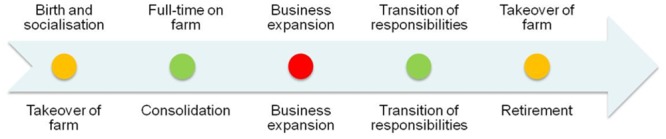
Farm generational model based on Burton [47]. The model highlights the dual life-cycle stages between an incumbent farmer (bottom) and their chosen successor (top). The five key life cycle stages are highlighted with the stages being colour coded to show the likelihood of change in the farming operation at each stage of the farmer’s life with red being high likelihood and green low likelihood.

The model implements a farmer/farm life-cycle (Fig 1) as discussed in Burton [47]. This life-cycle approach is used to incorporate the dynamics of succession within the model. Stages 1 and 5 instigate an assessment of the ongoing nature of the farm through an assessment of the likelihood of a successor joining the farming operation (Stage 1). Stage 5 will enable transition of the farm to the successor, although there is a process to enact the sale of the farm to a new or existing farmer agent if there is no successor by this stage.

If a successor is found in Stage 1, the likelihood is that the successor is groomed to operate the farm in a similar manner to the incumbent and life-cycle of the existing agent is reset to stage 1 to replicate the takeover of the farm by the successor. If a successor is not found, the farm is sold to a new or existing farmer agent. This disposal behaviour also provides an opportunity for a shift in land use in line with the preferences of the new owner. Constraining the decision-making process around a farm-based life-cycle process enables a more realistic expansion, succession, and disposal processes in relation to the farm to be examined and explored in relation to the wider land-use change that occurs.

### Social Network

When reviewing these social interactions from both a conceptual and theoretical perspective, it is obvious that farmer’s information networks are framed around their social interactions [18, 19, 48]. Distilling each of these networks further, they relate to two approaches that are implemented within the model: endorsements and imitation.

While endorsements and imitation within farming social networks are understood, the scale and impact that these process have on the decisions being undertaken difficult to quantify within a farming context. This provides challenges when defining the impact on an agent’s decision. Studies have found that the proximity to the people in your network is not as important as the stature of the person [48, 49]. The scale of impact for both processes within the ARLUNZ model is defined in line with expert opinion and experimentation, although with imitation an explicitly spatial process, we define imitation as having a reduced impact compared with endorsements.

### Endorsements

Endorsements are used in agent-based models to incorporate the transfer of qualitative knowledge. They capture a “subjective, but socially embedded agent's reasoning process about cognitive trajectories aimed at achieving information and preferential clarity over another, endorsed agent” [50, p. 1]. Endorsements work on the concept that information about a product, process or person (the endorsed) is transferred from one individual (the endorser) to another individual (the receiver) through a social process. The information that is transferred by the endorser is subjective and is then validated by the receiver based on the individual’s understanding of the endorser and the product, process or person.

Within the ARLUNZ model, the farmer agent will incorporate information on the success of the farming operation of the ten closest farmer agents who undertake the same enterprise as the farmer agent. Each farmer agent requests the profitability/ha of each of the farmer agents within their social network. Using these values, a mean profitability/ha value is derived for the farmer agent’s network and is then compared with farmer agent’s profitability/ha value. If the farmer agent’s profitability/ha is higher than the mean profitability/ha of the farmer agent’s social network, their likelihood of land use conversion is decreased by 0.1, making it less likely that the farmer agent will change land use. If lower, their likelihood of land use conversion is increased by 0.1, making it more likely that the farmer agent will change land use. The model assumes that the stature of each agent within the social network is equally weighted.

### Imitation

Imitation is an evolutionary process that helps agent populations observe and learn about the consequences of certain actions. The theory of Social Learning [51, 52] describes imitation as a process where an actor observes another actor being rewarded for understandable and reproducible behaviour. The original actor might then imitate that behaviour to try to achieve the same reward [53]. Imitation transfers knowledge through a one-way network, where information is ‘absorbed’ (potentially through both active and passive means) from the agent’s surroundings, and then used to inform the decisions they make. Farming is a visible activity and land use. The practice of farming is visible to all who passes by a farm, even more so to farmers in close proximity because of the regular exposure [54, 55]. Therefore we need to allow for imitation behaviour to play a role in shaping, informing or reinforcing the decisions that a farmer makes [54].

For the ARLUNZ model, the farmer agent will incorporate information from the farms that are geographically adjacent to their own farm regardless of the enterprise undertaken. If the economic component of the model proposes a change in land use, each farmer agent within their geographic network that does undertake the proposed land use is queried to return their profitability/ha value. Using these values, a mean profitability/ha value is derived for the farmer agent’s network and is then compared with farmer agent’s profitability/ha value. If the farmer agent’s profitability/ha is higher than the geographic network, their likelihood of land use conversion is decreased by 0.05, making it less likely they will change land use. If lower, the agent’s likelihood of land use conversion is increased by 0.05 making it more likely they will change land use.

## Behavioural constraints

There are two enterprise level behavioural constraints implemented within the model. The first is when a farmer agent adopts either the Forestry or Carbon Forestry (i.e., forests planted for the sole purpose of receiving payments for carbon sequestration) enterprise type. The farmer agent who selects either of these enterprises is constrained to it for the minimal length of the stand age required for the economically feasible production of wood, which equates to 25 years or 5 time steps of the model. This is implemented by reducing the farmer agent’s likelihood of conversion to 0% prior to the evaluation of the agent’s options.

The second enterprise level constraint is based on the proposed enterprise as identified by the economic component of the model. The costs of conversion and changes in the farmer agent’s lifestyle are not internalised in the model at this stage. Within the model we assume that the conversion costs and lifestyle changes between Sheep & Beef, Forestry, and Carbon Forestry are negligible. However, a conversion from one of these enterprises to Dairy (a significant up-front cost and change to their lifestyle) is only indirectly taken into account in the model. Consequently, if the model proposes a conversion to Dairy it reduces the farmer agent’s likelihood of conversion by 75% prior to the evaluation of their land use conversion options. This constraint accounts for the common economic and social costs that restrict conversions to Dairy in New Zealand.

## Model Parametrisation and Scenario

**Fig 2:**
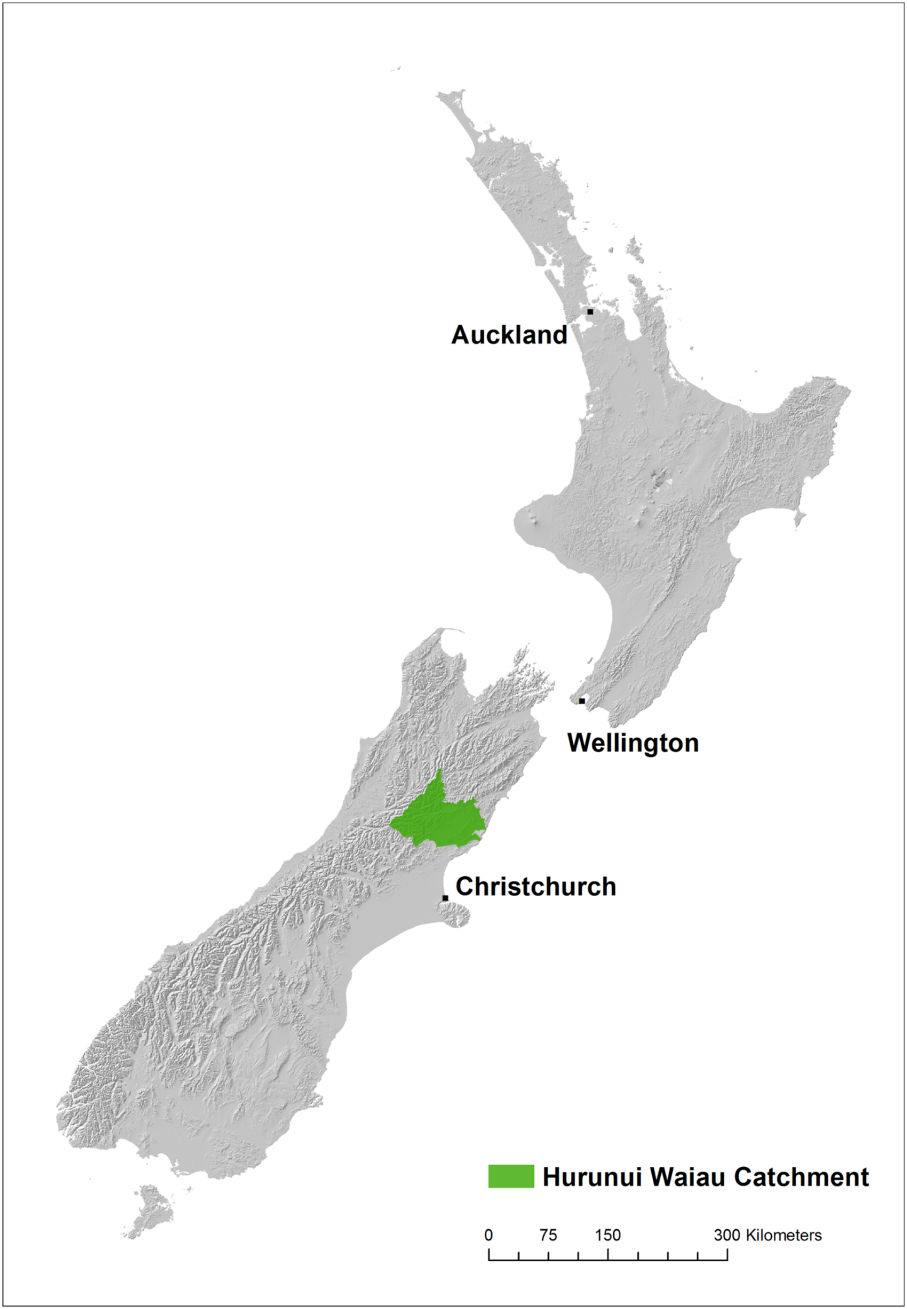
Location of the Hurunui and Waiau Catchments. The Hurunui and Waiau Catchments are located within the North Canterbury region of New Zealand’s South Island. The catchments are located in a region with a large and diverse set of land uses and agricultural enterprises and are also expecting significant changes in the land use.

We illustrate the utility of ARLUNZ to assess the economic and LULCC impacts to New Zealand’s forest and agricultural sector by simulating a GHG reduction policy on landowners in Hurunui and Waiau catchments in the North Canterbury region of New Zealand’s South Island (Fig 2). These catchments were selected because they are located in a region with a large and diverse set of land uses and agricultural enterprises and are also expecting significant changes in the land use. In addition to estimating changes in agricultural production, land use, and farm income, modelling a climate policy also allows us to assess the potential impacts on the catchment’s water quality through nutrient loading.

The data, parameters and assumptions within the model are as follows. The model covers a time horizon of 50 years with 10 incremental time steps within the model, each of which represent 5 years. The scenario assumes a real annual increase in farm commodity prices (milk, meat, and timber) of 2%, which is in line with the last 50 years of commodity prices. Forecasts from the Ministry for Primary Industries are closely aligned with our assumed price growth trajectories [1]. A succession success rate of 75% is implemented within the model. This value is in line with the expected rate as determined through a survey to assess the current state of succession planning on New Zealand farms [56]. Each agent starts with a probability of land use conversion of 0.2. However this is altered prior to each land use decision based on the information received through the social and geographic networks. After each farmer has undertaken a land use decision, the probability is reset to 0.2. This initial value was defined in line with the results found in a survey of New Zealand farmers which asked about the perceived likelihood of both land-use conversion over the subsequent 5 years [57]. Finally, the climate, and available technology (hence farm productivity), are held constant over the entire model simulation.

**Fig 3:**
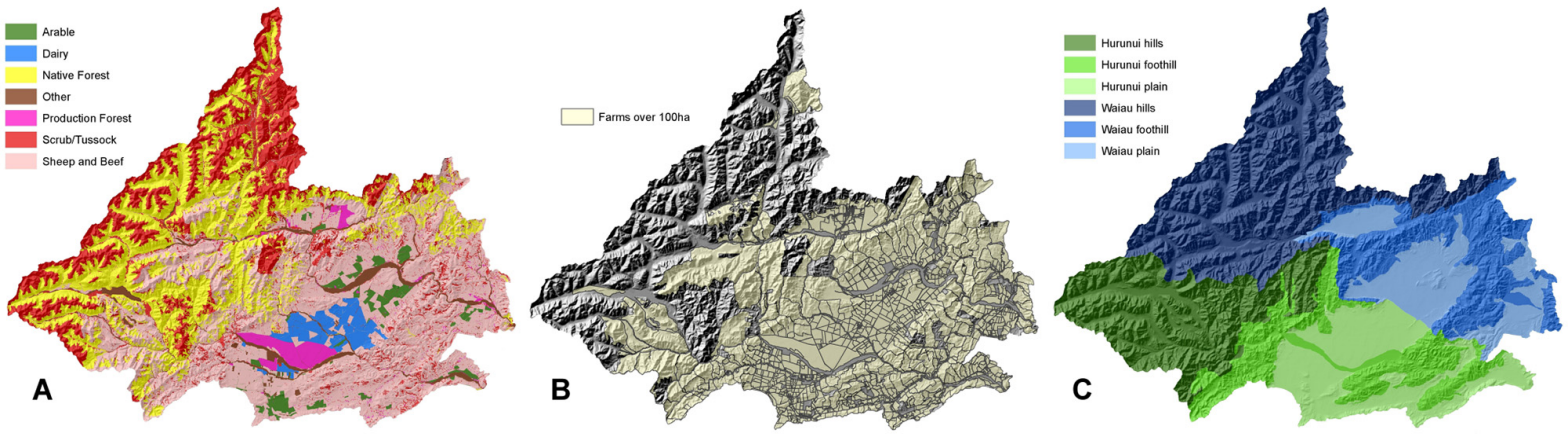
Detailed map of the Hurunui and Waiau catchments by (a) 2010 land use, (b) 2012 cadastral parcels, and (c) productivity zone. The data layers are used within the model to define the initial land use, farm locations and extents, and the expected productivity for each farm.

The land-use map used in the model was captured in June 2010 [44] (Fig 3a), and although the map includes seven different land uses, the model focuses on the four key enterprises that represent 94% of the productive land available within the catchment: Dairy, Sheep & Beef, Plantation Forestry, and Carbon Forestry. The cadastral boundaries used are from Land Information New Zealand [43] and represent the cadastral structure of the catchments as of August 2012 (Fig 3b), which was the closest database to the 2010 land use map. For this catchment, farmer agents are only created for farms in excess of 100 ha in size to focus on commercially operated enterprises and minimise the inclusion of lifestyle blocks in the model. Productivity zones are delineated by New Zealand’s Land Use Capability dataset [45] to define Plains (LUC Classes 1–4), Foothills (LUC Classes 5–6) and Hills (LUC Classes 7–8) within the catchment (Fig 3c). Any land owned by the Crown (e.g. native forest) is assumed to be non-productive in use and no farmer agents created [58].

The analysis focuses on two key aspects: the effect of GHG prices on LULCC, and the impact of LULCC through feedback effects from the farmers’ social networks. To simulate the effect of GHG prices on LULCC, we imposed a series of GHG prices on emissions (sequestration) from agricultural (forest) enterprises. The approach mimics the current implementation of forestry in the NZ Emissions Trading Scheme as well as the future implementation of the agricultural sector where the GHG price is imposed on the output produced by the farm (i.e., meat and milk solids).

We impose GHG prices between $0 and $60 NZD (approx. $0 and $50 USD) per tonne carbon dioxide equivalent (tCO2e) to provide a range of possible outcomes based on the realised GHG price. This range of GHG prices are in line with projections published by New Zealand’s Ministry for the Environment [59]. To simulate the impact of feedback effects from the social networks, we compared the impacts of farmer agents utilising, and not utilising, information received through their social networks within their land use conversion decisions. The model was run using 50 random seed values for each of the scenarios being implemented to provide a sample that encompasses the range of scenario outcomes. In total there were 700 model runs undertaken for this analysis.

## Results

The average catchment-level estimates for key outputs from the different model simulations are listed in Table 1. Total farm net revenue for the catchments is estimated to increase over time, regardless of the GHG price, as a result of increasing commodity prices and landowners switching to more profitable enterprises. For the no GHG price baseline, net revenue is estimated to increase from $173 million in 2015 to $660 million in 2055 (annual growth rate of about 3.4%). Imposing a GHG price policy reduces farm net revenue by about 1–2% over the 50 year time horizon, depending on the scenario. However, more immediate effects could see reduction between 7–13% in early periods as landowners have yet to fully adjust to the policy shock.

**Table 1:** Mean values for key model outputs across the no GHG price baseline and the $20, $40, and $60 tCO2e GHG prices.

GHG Price		Farm Net Revenue	GHG Emissions	Net GHG Emissions	N Leaching	P Loss
($/tCO2e)		($M NZD)	(MtCO2e)	(MtCO2e)	(tN)	(tP)
*2015*						
$0	*Mean*	172.6	0.991	0.628	4,440	38.0
	*95% CI*	(171.3, 173.8)	(0.983, 0.998)	(0.593, 0.664)	(4408, 4471)	(37.7, 38.3)
$20	*Mean*	160.5	0.992	0.627	4,355	37.3
	*95% CI*	(159.0, 162.0)	(0.984, 0.999)	(0.595, 0.660)	(4325, 4385)	(37.0, 37.6)
$40	*Mean*	149.5	0.984	0.593	4,338	37.0
	*95% CI*	(147.2, 151.7)	(0.975, 0.992)	(0.553, 0.633)	(4306, 4370)	(36.7, 37.3)
$60	*Mean*	149.7	0.979	0.441	4,338	36.8
	*95% CI*	(145.3, 154.1)	(0.970, 0.987)	(0.382, 0.500)	(4299, 4378)	(36.5, 37.2)
*2035*						
$0	*Mean*	360.9	1.081	0.352	5,989	45.1
	*95% CI*	(356.6, 365.3)	(1.070, 1.091)	(0.307, 0.396)	(5918, 6060)	(44.6, 45.6)
$20	*Mean*	358.3	1.067	0.296	5,942	44.2
	*95% CI*	(353.5, 363.0)	(0.156, 1.077)	(0.245, 0.347)	(5870, 6013)	(43.7, 44.7)
$40	*Mean*	355.7	1.060	0.255	5,957	44.1
	*95% CI*	(351.0, 360.4)	(1.050, 1.071)	(0.201, 0.310)	(5892, 6022)	(43.6, 44.6)
$60	*Mean*	356.8	1.042	0.140	5,878	43.3
	*95% CI*	(350.6, 363.0)	(1.033, 1.052)	(0.091, 0.190)	(5804, 5952)	(42.9, 43.8)
*2055*						
$0	*Mean*	660.3	1.176	0.323	7,320	52.1
	*95% CI*	(651.6, 668.9)	(1.163, 1.189)	(0.278, 0.367)	(7222, 7417)	(51.5, 52.8)
$20	*Mean*	659.1	1.166	0.286	7,264	51.3
	*95% CI*	(650.3, 667.8)	(1.153, 1.179)	(0.231, 0.341)	(7168, 7359)	(50.7, 52.0)
$40	*Mean*	658.4	1.152	0.206	7,263	50.9
	*95% CI*	(649.0, 667.8)	(1.138, 1.166)	(0.154, 0.258)	(7157, 7370)	(50.2, 51.6)
$60	*Mean*	649.6	1.131	0.130	7,156	49.9
	*95% CI*	(639.9, 659.3)	(1.117, 1.144)	(0.081, 0.180)	(7050, 7262)	(49.2, 50.6)

Catchment-wide net GHG emissions (livestock emissions less forest carbon sequestration) are estimated to decline over time, even in the baseline. This is due to an expansion of Forestry in the less productive hills region, which is discussed in more detail below. Gross (livestock) GHG emissions do increase over time as dairy herds increase; however, this increase is small, about 0.4%/yr, as most of the Dairy area is expected to come from the conversion of already high-emitting Sheep & Beef enterprises. N leaching and P loss are both estimated to increase over time for all modelled scenarios, again due to the expansion of Dairy. In all scenarios, the annual increase in nutrients is about 1%/yr, suggesting that policy instrument focusing on GHG price alone may not have much of an impact on reducing other environmental outputs in the study area.

## Land Use

**Fig 4:**
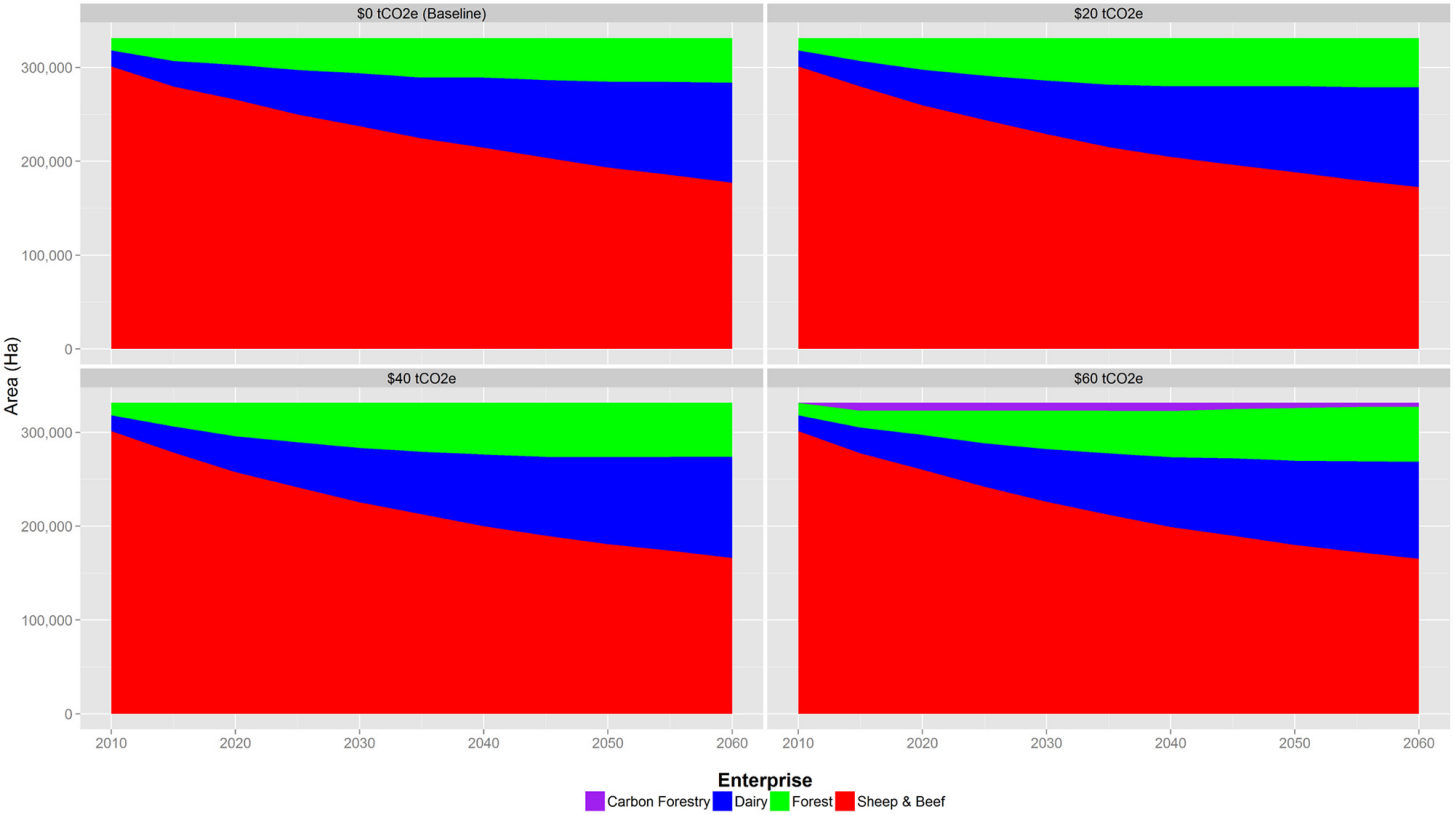
Change in land use for $0 to $60/tCO2e GHG price scenarios. For all scenarios, the area of both Dairy and Forest enterprises will increase over time. While Sheep & Beef will still be the main land use across all scenarios, the area it covers reduces significantly. Mean values, standard deviation, and confidence intervals for this figure are available in S1 Table.

We project that the area of both Dairy and Forest enterprises will increase over time in both the baseline and all of the GHG price scenarios (Fig 4). In the baseline, mean Dairy area increases from the initial 16,900 ha to 106,472 ha over the 50-year period (with a 95% Confidence Interval (95% CI) of 1,899 ha). Although expansion this seems large, it is not unrealistic. First, the area of Dairy land in Canterbury increased by 172% between 1996 and 2008, and it is projected to expand by an additional 51% by 2020 [46]. Second, the Hurunui and Waiau catchments have already witnessed additional conversion to Dairy over the last 5 years, as forests in the highly-productive flat areas of the catchment reach harvest age. Third, there are ongoing discussions of implementing the Hurunui Water Project, which would expand the area of irrigated land by an additional 41,500 ha, bringing the total irrigated area of the Hurunui and Waiau catchments to over 72,000 ha [60]. Therefore our projections are within what may be expected to occur in this catchment over the next 50 years.

The mean area of pine plantations are also estimated to increase in the baseline over the projected period, totalling 47,608 ha in 2060 (95% CI of 2,263 ha), up from 13,180 ha in 2010. This is because in parts of the catchment, forestry can be more profitable than Sheep & Beef, but not have the capability to sustain a Dairy operation. As a result, mean area for Sheep & Beef is reduced from 301,245 ha in 2010 to 177,245 ha by 2060 (95% CI of 2,906 ha).

GHG prices further incentivise the expansion of forestry, and increases in GHG price. This is because landowners face no GHG emissions price on land where they conduct rotational forestry, whereas all livestock operations do. Therefore while they are not receiving payments for carbon sequestration, they are also not facing any penalty for producing GHG emissions on the land on which they are no longer grazing livestock. The model estimates that Carbon Forestry (i.e. native tree plantations that receive payment for permanently sequestering carbon and not harvested) is not implemented until GHG prices reach $60/tCO2e. This is because pine plantations are accruing greater profits under most GHG prices even if those forest owners are not receiving payments for carbon sequestration (i.e. the timber is worth more than the carbon).

Forest area, which expands in all scenarios, does so primarily through the conversion from Sheep & Beef, which loses 3–6% of its area depending on the GHG price scenario. The area of Dairy is estimated to fluctuate between increasing by about 2% on average relative to the baseline in the $20/tCO2e scenario to declining by up to 3% in the $60/tCO2e scenario. This suggests that at lower prices, some landowners may be incentivised by the relative increases in net revenue compared to Sheep & Beef and thus convert their farm, while higher prices do not have the same effect.

Total land-use change in the catchments over the course of the model simulation period is expected to be most dramatic for the high GHG price scenario ($60/tCO2e). For this scenario we estimate that in 2060, the area of Dairy will be 103,203 ha (95% CI of 2,139 ha), Sheep & Beef at 165,333 ha (95% CI of 2,768 ha), and Forests (both Production and Carbon) at 62,789 ha (95% CI of 2,082 ha).

## Farm Net Revenue

**Fig 5:**
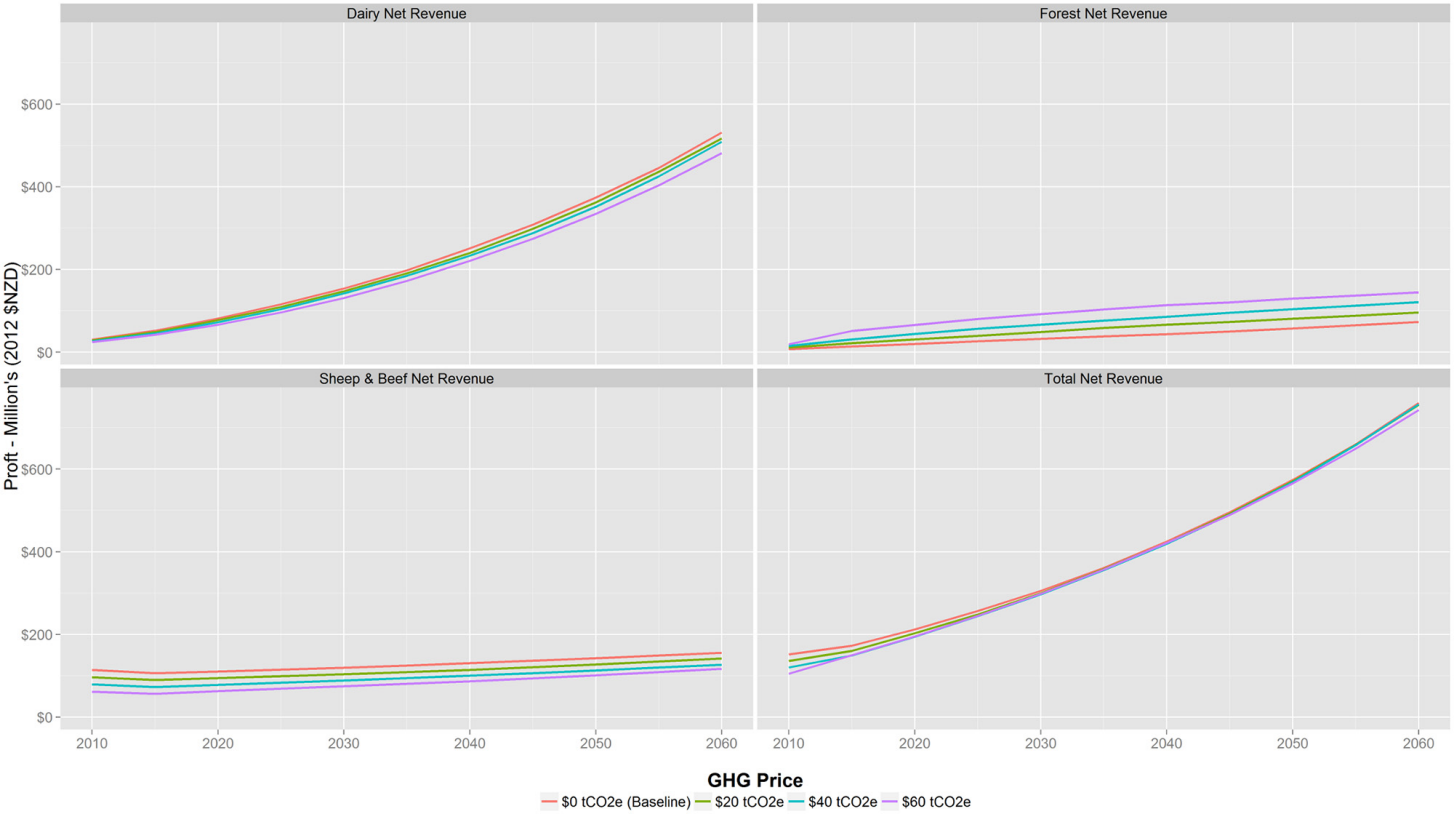
Change in enterprise net revenue for $0 to $60/tCO2e GHG price scenarios. Farm net revenue is estimated to increase over time regardless of the GHG price. This is the result of the 2% commodity price increase assumption and increased conversion to Dairy. Mean values, standard deviation, and confidence intervals for this figure are available in S1 Table.

Farm net revenue is estimated to increase over time regardless of the GHG price. This is primarily because of 2% commodity price increase assumption and expansion in Dairy farms in the catchment (Fig 5). Total dairy net revenue increase from $52 million in 2015 to between $481 and $531 million in 2060 (95% CI of $11 million), with the largest increases occurring under the baseline. The range of net revenue for Forest and Sheep & Beef is more variable over the different GHG price scenarios, reflecting the range of land-use change between the two types. For example, Sheep & Beef net revenue could increase from $107 million in 2015 to between $117 and $156 million in 2060 (95% CI of $3 million), depending on the GHG price. In terms of forestry, net revenue is estimated to increase from $14 million in 2015 to between $73 and $145 million in 2060 (95% CI of $5 million), and the payments for carbon sequestration that encourage Carbon Forestry in the $60/tCO2e scenario further enhance profitability from forestry.

## Greenhouse Gas Emissions

**Fig 6:**
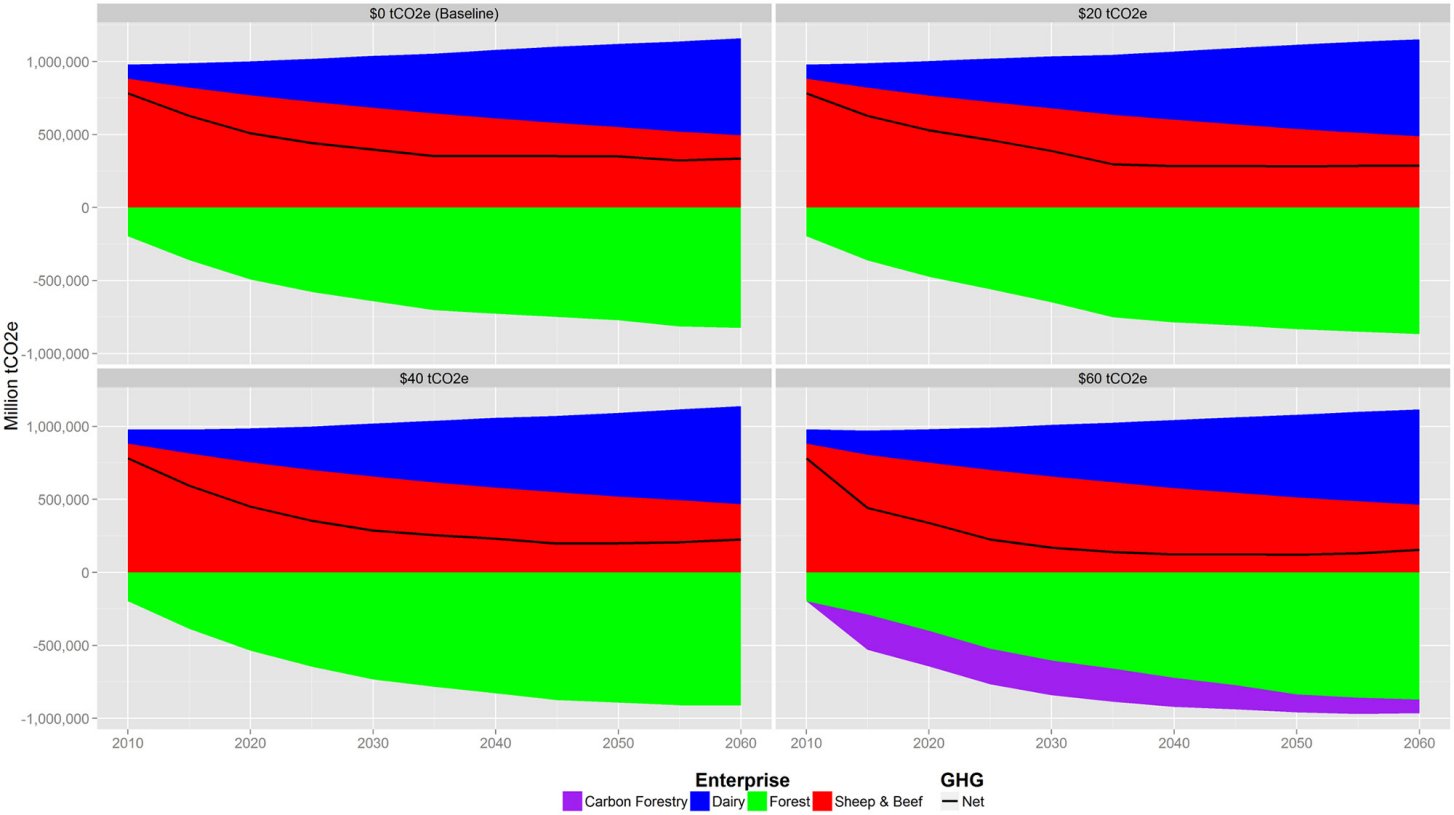
Change in GHG emissions by enterprise for $0 to $60/tCO2e GHG price scenarios. For all scenarios, the total net GHG emissions are estimated to decrease over time, including the $0/tCO2e baseline. Most decreases are due to expanding forest area, which results in larger net reductions than only gross reductions from livestock emissions. Mean values, standard deviation, and confidence intervals for this figure are available in S1 Table.

**Fig 7:**
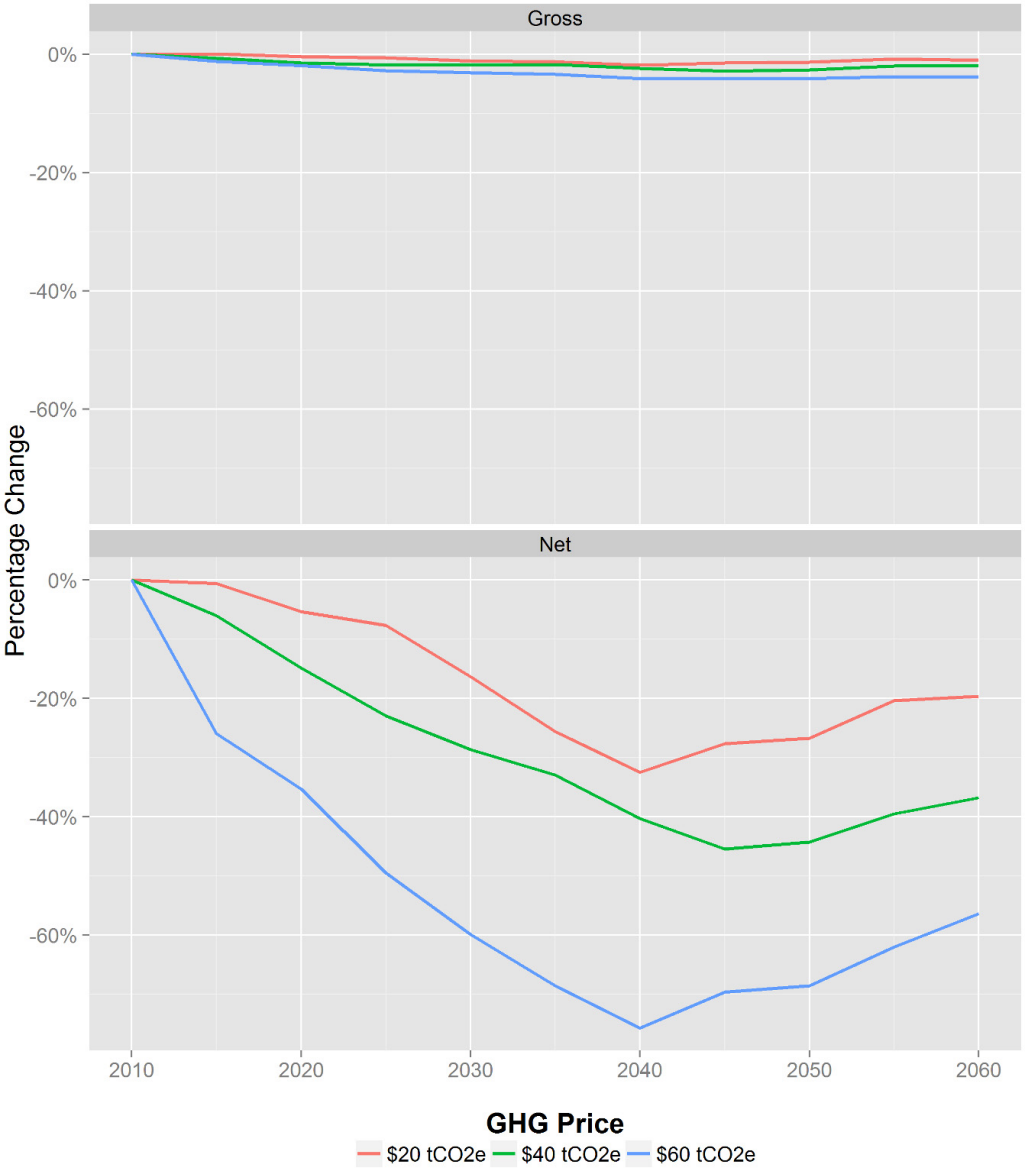
Relative change from the $0/tCO2e baseline in gross (top) and net (bottom) GHG emissions. In the $60/tCO2e GHG price scenario, net GHGs reduce to about 0.23 MtCO2e/yr in 2060, a reduction of about 58% against the baseline. Peak reduction occurs in 2040 just prior to the first wave of forests planted in the first time step being converted to another land use. Mean values, standard deviation, and confidence intervals for this figure are available in S1 Table.

Total net GHG emissions are estimated to decrease over time, even for baseline where they go from about 0.78 in 2015 to 0.34 MtCO2e/yr (95% CI of 0.05 MtCO2e) in 2060 (Fig 6). As expected, both net and gross emissions decrease with GHG price. Most decreases are due to expanding forest area, which results in larger net reductions than only gross reductions from livestock emissions. In the ‘high’ GHG price scenario where emissions are taxed at $60/tCO2e, net GHGs reduce to about 0.15 MtCO2e/yr in 2060 (95% CI of 0.05 MtCO2e), a reduction of about 54% relative to the baseline (Fig 7). The same policy results in gross emissions being reduced by just 4% relative to the baseline case.

The lack of significant change in livestock emissions despite quantified land use change suggests that the GHG price policies are incentivising landowners to make more efficient use of their land. That is, they are shifting stock to areas that are most productive and then offsetting these emissions through afforestation elsewhere. Therefore they can reduce the area that is grazed without reducing overall herd size.

## Social and Geographic Network Effects

As discussed above, the relative profitability of Dairy could potentially catalyse large land use change in the catchments over the simulation period, regardless of the GHG price or feedback effect. Isolating the feedback effect by only focusing on the estimates where we allow for network effects, we see an even larger shift in land use.

Table 2 highlights how network effects increased the conversion of enterprises to Dairy in the two productivity zones where it is feasible to do so (i.e., Plains and Foothills). The network effects impact on conversion into Dairy is the most pronounced in the baseline ($0/tCO2e) but can be seen throughout all GHG pricing options. Over the various GHG price scenarios, network effects increase the mean proportion of Plains Dairy land (relative to total catchment area) by between 2.4 and 3.7% and the proportion of Foothills Dairy land increases by between 8.5 to 11.9%.

**Table 2:** Social and geographic network effects on the proportion of land use by productivity zone for the no GHG price baseline and the $20, $40, and $60 tCO2e GHG prices.

$0/tCO2e (Baseline)	%Land Use Network Off	%Land Use Network On	Change	$20/tCO2e	%Land Use Network Off	%Land Use Network On	Change
Plains | Forestry	3.3%	1.4%	-1.9%	**Plains | Forestry**	2.9%	1.7%	-1.2%
Plains | Sheep & Beef	27.0%	25.2%	-1.8%	**Plains | Sheep & Beef**	27.2%	25.9%	-1.3%
Plains | Dairy	7.1%	10.7%	3.6%	**Plains | Dairy**	7.3%	9.8%	2.5%
Foothills | Forestry	1.0%	0.5%	-0.5%	**Foothills | Forestry**	1.0%	0.8%	-0.2%
Foothills | Sheep & Beef	38.6%	27.8%	-10.8%	**Foothills | Sheep & Beef**	37.6%	29.5%	-8.1%
Foothills | Dairy	4.3%	16.2%	11.9%	**Foothills | Dairy**	5.0%	13.5%	8.5%
Hills | Forestry	16.4%	16.4%	0.0%	**Hills | Forestry**	16.4%	16.4%	0.0%
Hills | Sheep & Beef	2.3%	1.6%	-0.7%	**Hills | Sheep & Beef**	2.6%	2.4%	-0.2%
Hills | Dairy	0.0%	0.0%	0.0%	**Hills | Dairy**	0.0%	0.0%	0.0%
**$40/tCO2e**	**%Land Use Network Off**	**%Land Use Network On**	**Change**	**$60/tCO2e**	**%Land Use Network Off**	**%Land Use Network On**	**Change**
Plains | Forestry	2.4%	1.7%	-0.7%	**Plains | Forestry**	3.3%	2.9%	-0.4%
Plains | Sheep & Beef	27.1%	24.2%	-2.9%	**Plains | Sheep & Beef**	27.2%	25.2%	-2.0%
Plains | Dairy	7.8%	11.5%	3.7%	**Plains | Dairy**	6.9%	9.3%	2.4%
Foothills | Forestry	0.9%	0.9%	0.0%	**Foothills | Forestry**	1.0%	1.0%	0.0%
Foothills | Sheep & Beef	37.9%	27.4%	-10.5%	**Foothills | Sheep & Beef**	38.2%	30.7%	-7.5%
Foothills | Dairy	5.3%	15.3%	10.0%	**Foothills | Dairy**	4.4%	12.9%	8.5%
Hills | Forestry	16.4%	16.4%	0.0%	**Hills | Forestry**	16.4%	16.4%	0.0%
Hills | Sheep & Beef	2.1%	2.6%	0.5%	**Hills | Sheep & Beef**	2.6%	1.6%	-1.0%
Hills | Dairy	0.0%	0.0%	0.0%	**Hills | Dairy**	0.0%	0.0%	0.0%

In all scenarios, the proportion of catchment area used for Dairy farms becomes larger in the Foothills than the Plains when network effects are active. This is primarily attributed to an increased variation in relative profitability of Dairy in the Foothill productivity zone, and the successful Dairy farmers influencing the conversion other farmer agents to Dairy through social and geographic network effects.

**Fig 8:**
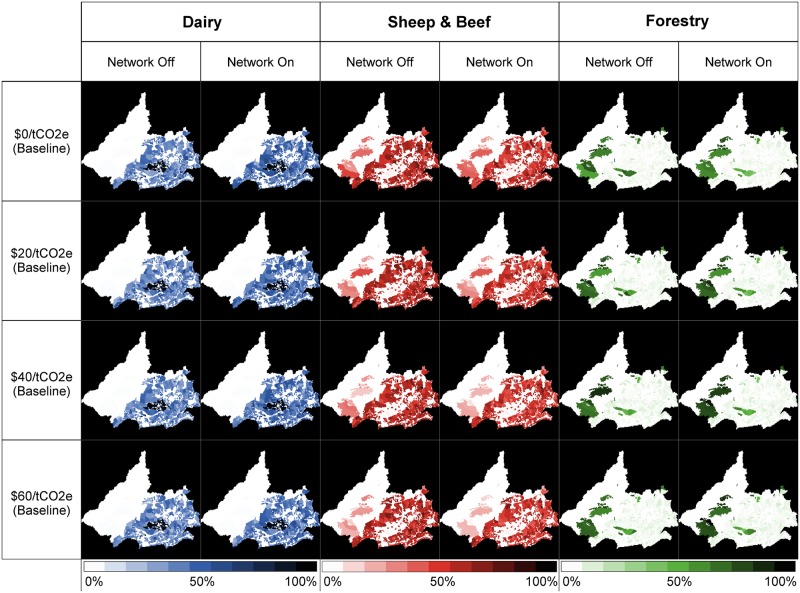
Changes in the spatial arrangement of three enterprises (Dairy, Sheep & Beef, and Forestry) caused by network effects and the GHG prices. Darker colours (Blue, Red, and Green respectively) represent a higher likelihood of the farm being used in the enterprise at the end of the combined model runs. For further analysis, the individual images are available in S1 File.

These aspects are reinforced when exploring the resulting land use maps at the conclusion of each model run. Analysing at a farm parcel level, we developed a probability that the farm will result in each of the three main enterprises, across the four GHG prices, and network effects. Fig 8 highlights that regardless of the GHG price network effects amplify the financial benefits of the Dairy enterprises. This results in the clustering of the enterprise within the Plains and Foothills productivity zones. This amplification is reversed for Sheep & Beef while GHG prices play stronger role in the likelihood of a farm resulting in a Forestry enterprise.

Conversion to Forestry in the Hill productivity zone appears to be unaffected by the role of network effects. This is because the enterprise utilises almost all of the Hills regardless of the policy scenario or assumptions about network effects, as it is more profitable over the long run than Sheep & Beef. A more interesting aspect is the remaining forestry land within the highly productive Plains. As seen in Fig 4a, there is a significant amount of Forestry at the initial state of the model. In all scenarios, the network effects reduced the amount of Forestry land which is converted into Dairy. In the $40/tCO2e and $60/tCO2e scenarios, the network effect of this shift is reduced because of the financial impacts of a high GHG price. These aspects highlight how minor pull/push forces within a farmer’s social network can have a cumulative social effect within the wider farming community, in particular around the spatial arrangement of the land use change.

## Conclusions

This paper utilised an agent-based model to highlight the spatial, network, environmental, and economic effects of GHG price policies on an agricultural landscape within New Zealand. From an economic perspective, the model highlights that a GHG price on land-based emissions would lead to reductions in the level of GHGs produced through a combination of emissions reductions and increased forest carbon sequestration. Even with these changes, net revenue at catchment level actually increases over simulation period because payments for forest carbon sequestration more than offset the increased costs on Sheep & Beef and Dairy enterprises. While the model currently only allows for a single enterprise to be enacted on a farm, it would be expected that farmers who are willing to become at least part-time foresters would see increased level of net revenue. While social and geographic network effects do not have a large effect on the overall net revenue or environmental outputs for the catchments, they do have an effect in the spatial arrangement of enterprises, which could in the future provide input into associated environmental aspects such as freshwater pollution.

From a land use perspective, the model showed that there will be substantial land-use change likely to occur over the next 50 years. Dairy expanded in size across all scenarios because of high relative net revenue. Forestry more than doubled in size by 2060 for all GHG price scenarios through a combination of better utilisation in the Hills productivity zone, and the increase in relative net revenue with higher GHG prices. Sheep & Beef contracts in line with GHG price increases because of low net revenue and high GHG emissions rates.

The model estimated that although the total gross GHG emissions in the catchment increase over time, the total net GHG emissions in the catchment are estimated to decline regardless of the policy scenario. For example, while livestock emissions are estimated to increase by a rate of about 0.5%/yr between now and 2060, net emissions are estimated to decline by 1–2%/yr over the same period. This is primarily due to an expansion of Forestry in the Hills region, which significantly increases the level of carbon sequestration in the catchments. In contrast, we estimated the large conversion of Sheep & Beef to Dairy could cause N leaching and P loss to increase over time for all modelled scenarios at a rate of about 1%/yr. This suggests that GHG price policy alone may not have much of an impact on reducing other environmental outputs in the study area. We note that while the Hurunui-Waiau catchment is an important agricultural region, the impacts may differ in other parts of New Zealand.

The use of a coupled economic and agent-based approach provides a range of options for future research. Recent research into farming behaviour within New Zealand [57] highlights the fragmented practice of farming caused through the process of diversification with over 50% of farms having 2 or more enterprises on their farm. Future development will enable a heterogeneous application of enterprises within an individual farm so we can explore a more realistic application of land use and the resulting economic and environmental aspects.

We recognise that the model currently only explores the complete farm conversion from enterprise to enterprise. However the application of within-farm management options (such as reducing stocking rates, fencing streams, or planting riparian buffers) could also have significant economic and environmental effects. Future research will develop and explore the application of these within-farm management options, the sensitivity of the model to commodity price changes, the uptake through social and geographic networks (in particular the role that stature has within social networks), and model the wider effects within a revised model. To do this we will require detailed farm-level surveys to underpin the model but the approach would also provide additional insight on defining farmer types, social networks, environmental values and their willingness to adopt new technologies to adapt to climate change and commodity price risk.

## Supporting Information

S1 File Individual high resolution images showing changes in the spatial arrangement of three enterprises (Dairy, Sheep & Beef, and Forestry) caused by network effects and the GHG prices. (ZIP)

S1 Table Mean values, standard deviation, and confidence intervals for Figs 4–7. (XLSX)
