# Supplementary figures and images for: Estimating Impacts of Climate Change Policy on Land Use: An Agent-Based Modelling Approach
Source: PLoS One. 2015 May 21;10(5):e0127317. doi: 10.1371/journal.pone.0127317 (PMC4440817; doi:10.1371/journal.pone.0127317)

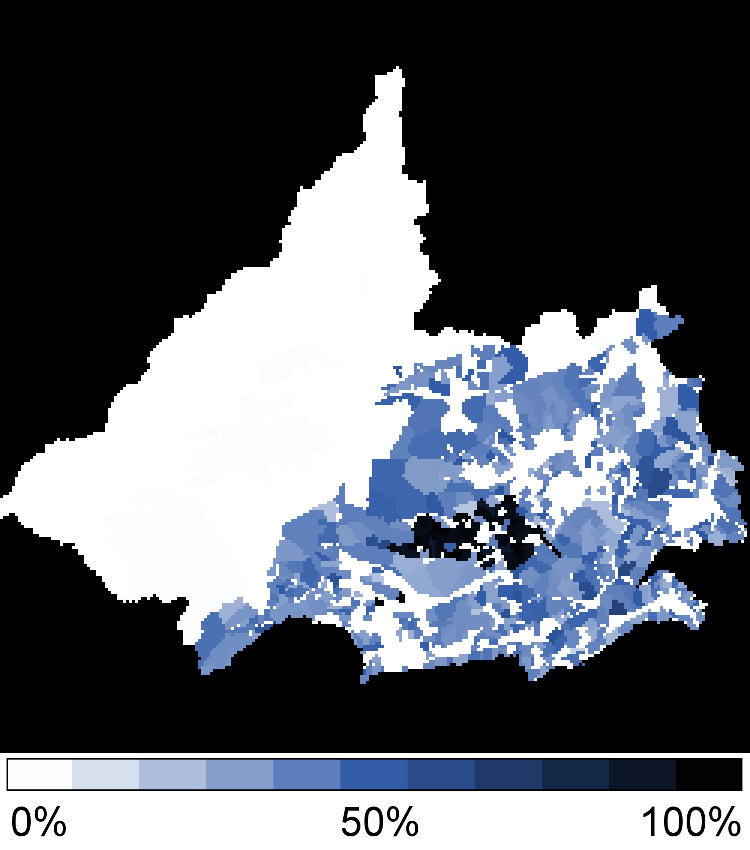

Supplement: S1 File — (ZIP) [file pone.0127317.s001.zip › ARLUNZ_Dairy_GhG_0_NetworksOff.png]

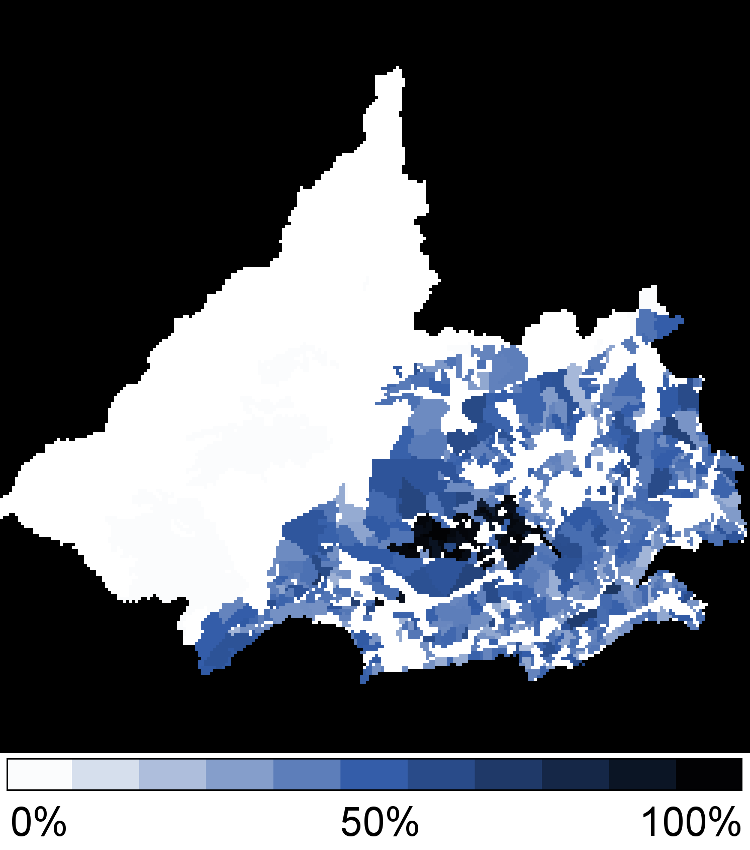

Supplement: S1 File — (ZIP) [file pone.0127317.s001.zip › ARLUNZ_Dairy_GhG_0_NetworksOn.png]

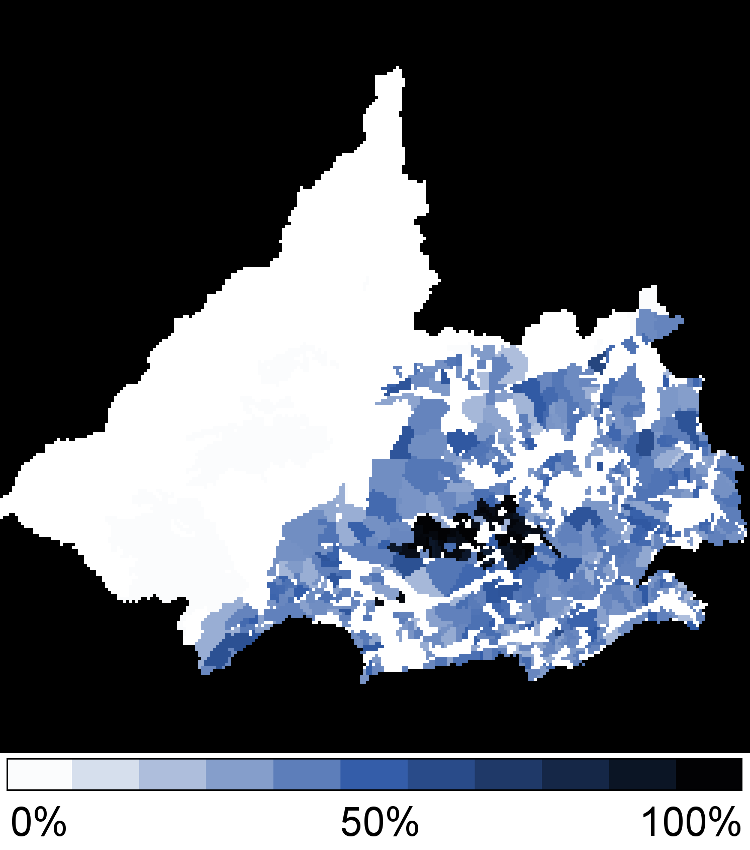

Supplement: S1 File — (ZIP) [file pone.0127317.s001.zip › ARLUNZ_Dairy_GhG_20_NetworksOff.png]

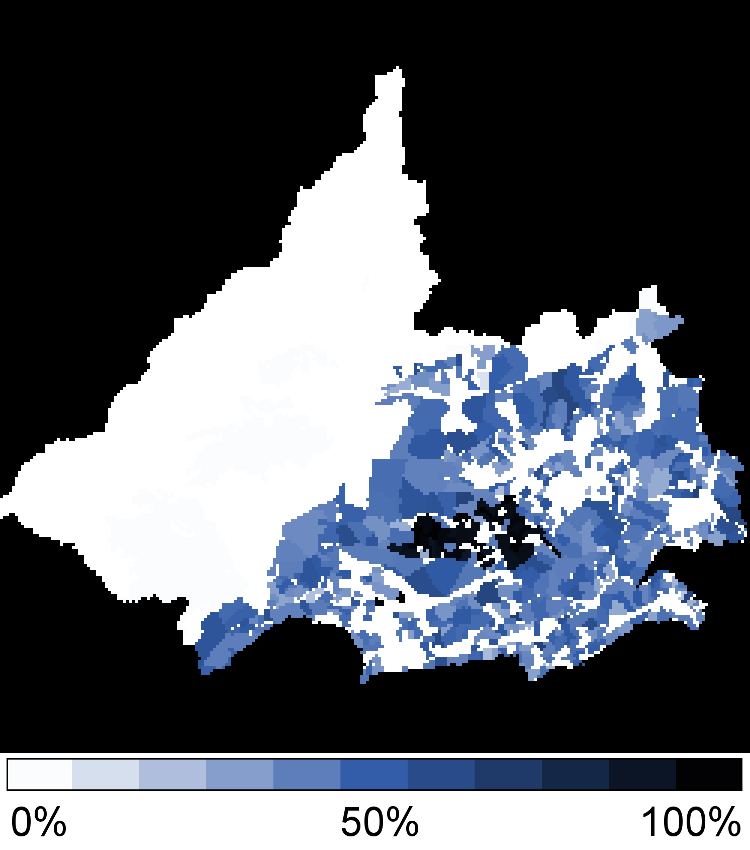

Supplement: S1 File — (ZIP) [file pone.0127317.s001.zip › ARLUNZ_Dairy_GhG_20_NetworksOn.png]

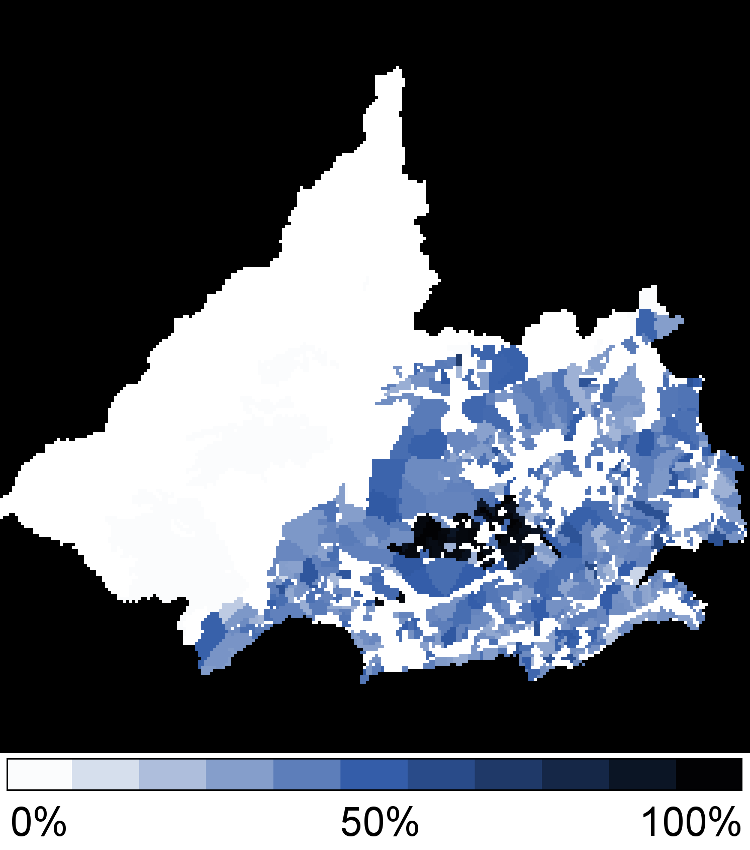

Supplement: S1 File — (ZIP) [file pone.0127317.s001.zip › ARLUNZ_Dairy_GhG_40_NetworksOff.png]

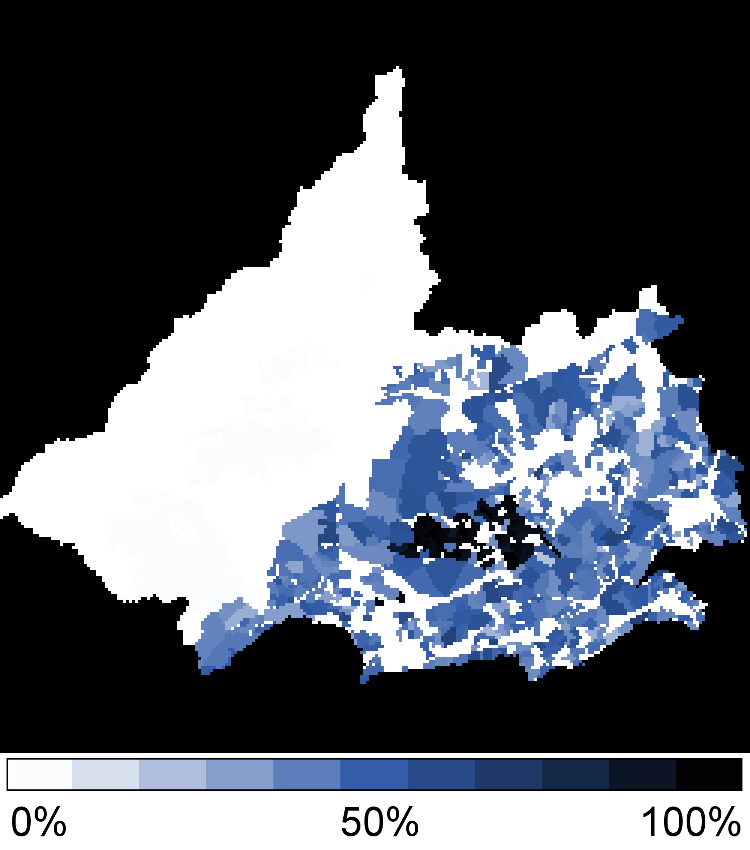

Supplement: S1 File — (ZIP) [file pone.0127317.s001.zip › ARLUNZ_Dairy_GhG_40_NetworksOn.png]

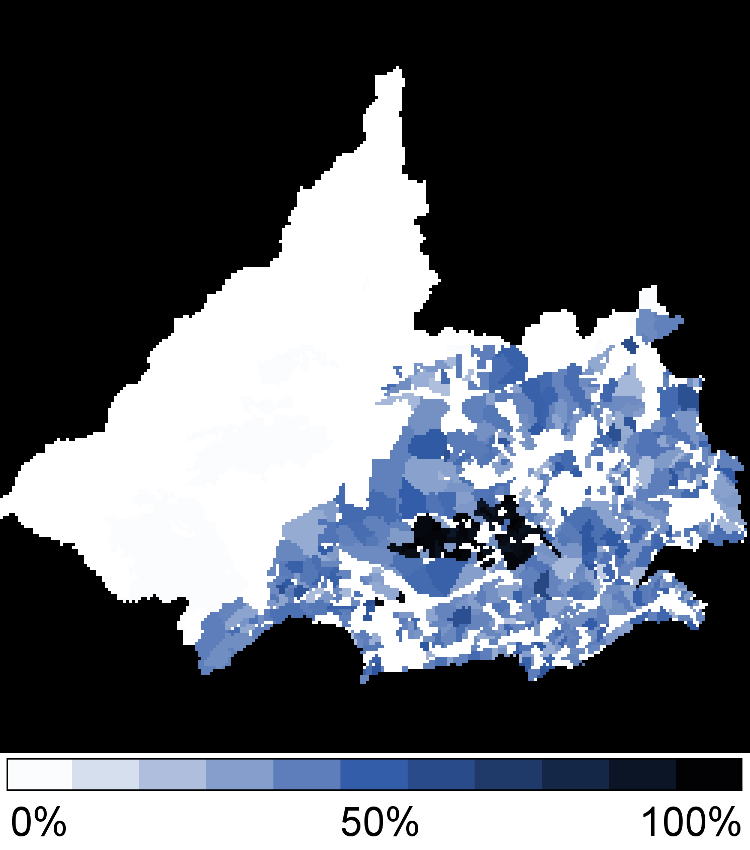

Supplement: S1 File — (ZIP) [file pone.0127317.s001.zip › ARLUNZ_Dairy_GhG_60_NetworksOff.png]

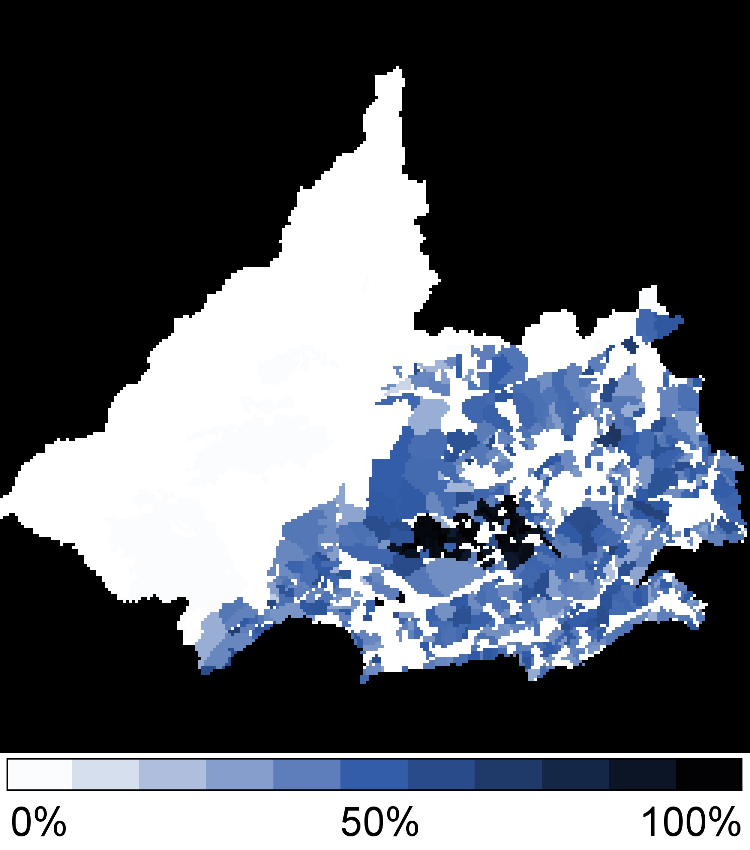

Supplement: S1 File — (ZIP) [file pone.0127317.s001.zip › ARLUNZ_Dairy_GhG_60_NetworksOn.png]

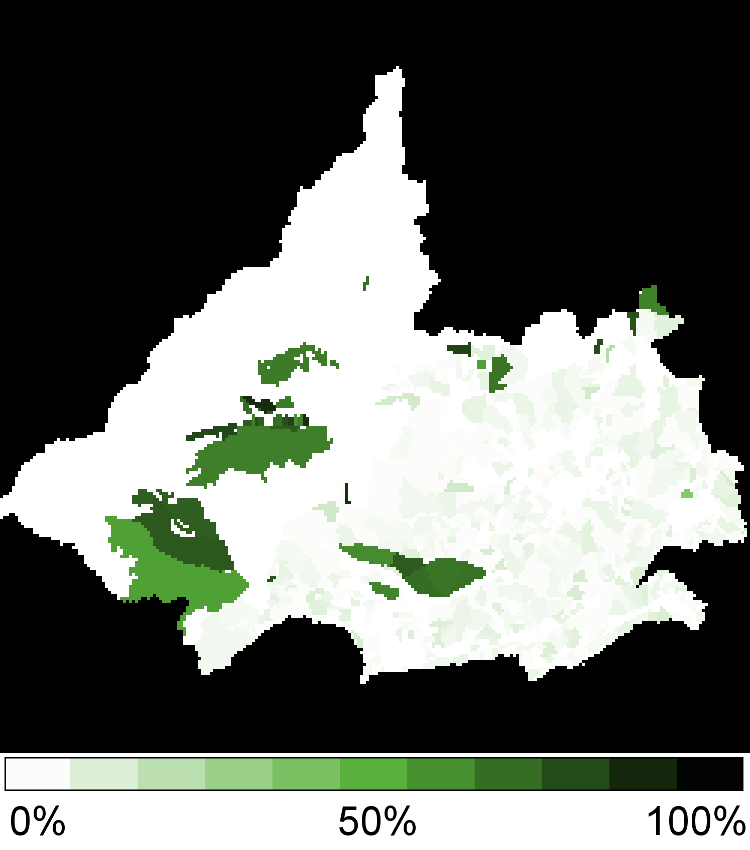

Supplement: S1 File — (ZIP) [file pone.0127317.s001.zip › ARLUNZ_Forestry_GhG_0_NetworksOff.png]

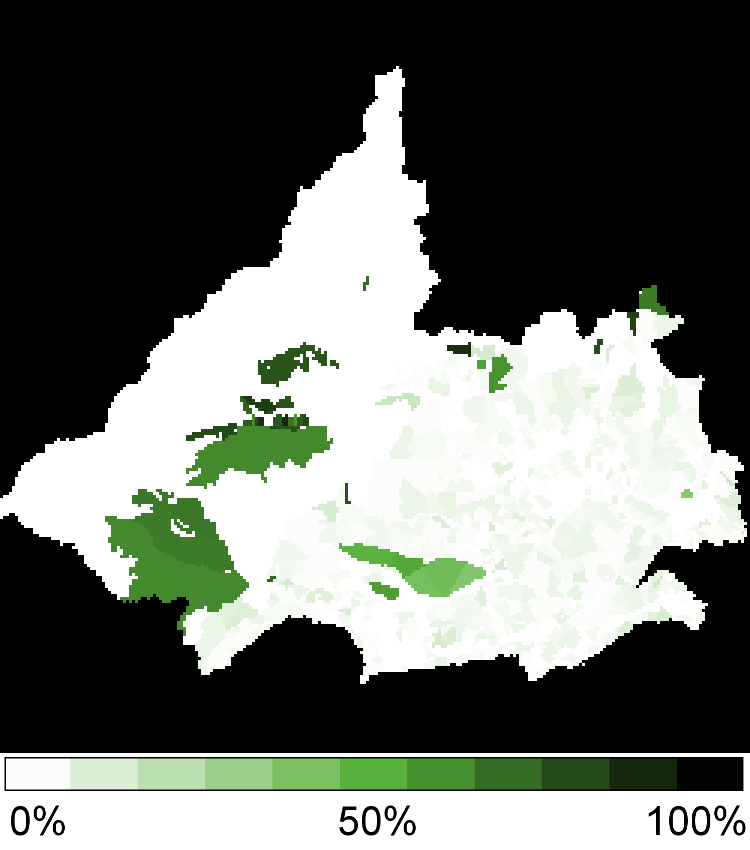

Supplement: S1 File — (ZIP) [file pone.0127317.s001.zip › ARLUNZ_Forestry_GhG_0_NetworksOn.png]

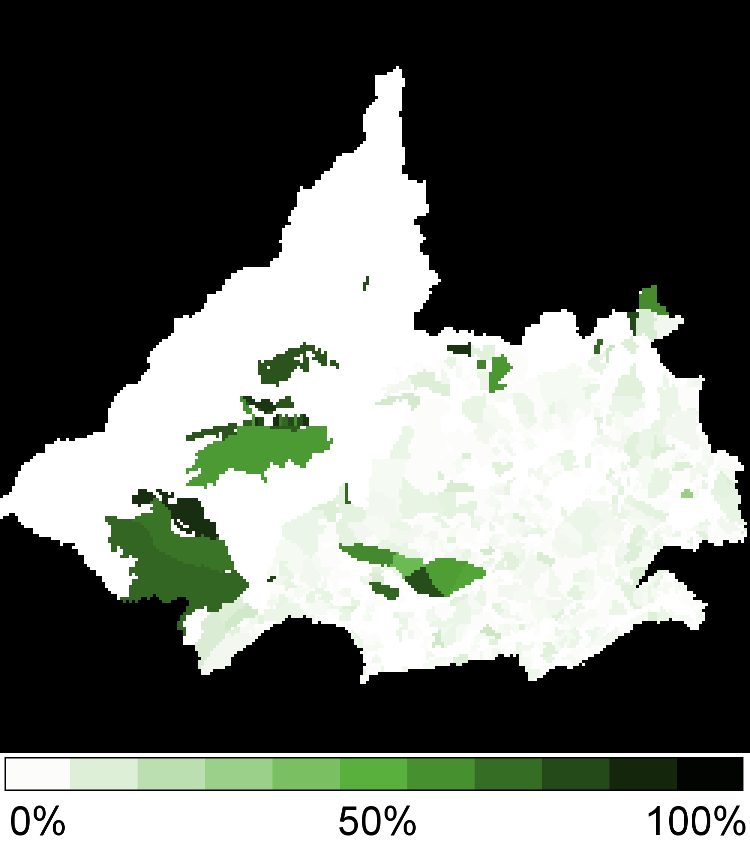

Supplement: S1 File — (ZIP) [file pone.0127317.s001.zip › ARLUNZ_Forestry_GhG_20_NetworksOff.png]

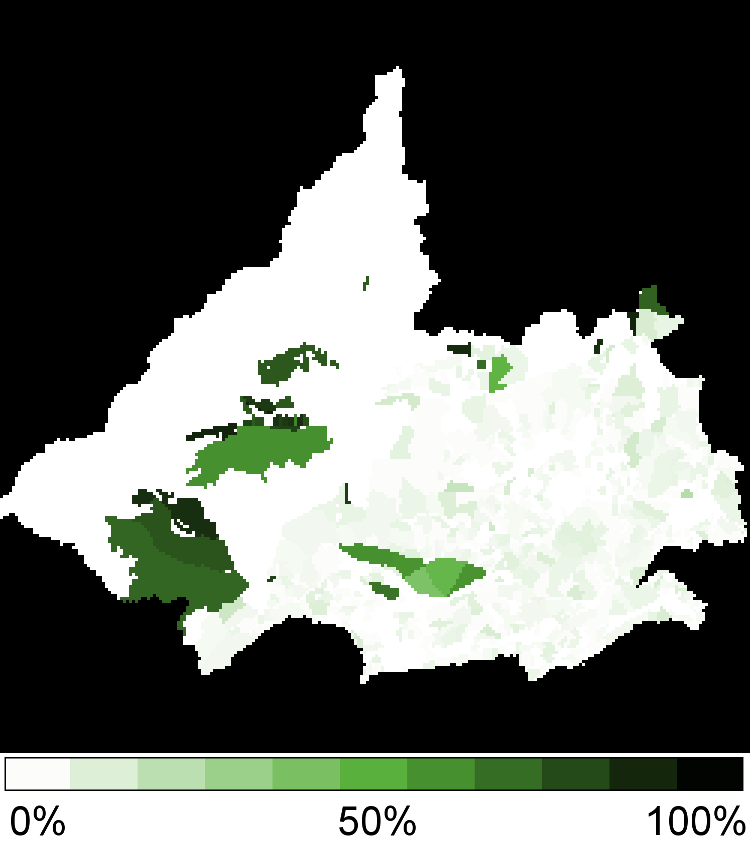

Supplement: S1 File — (ZIP) [file pone.0127317.s001.zip › ARLUNZ_Forestry_GhG_20_NetworksOn.png]

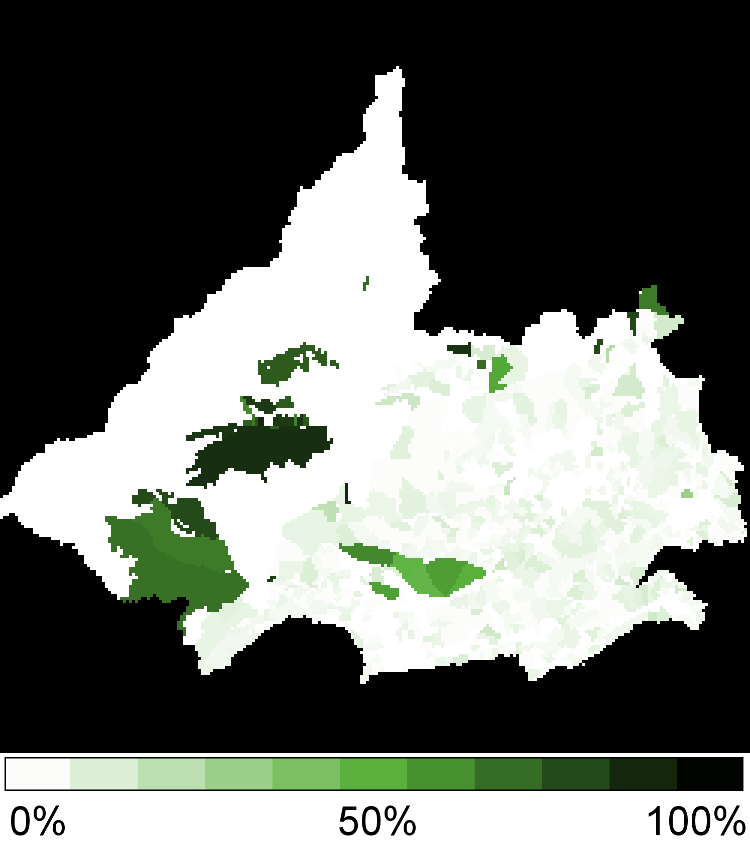

Supplement: S1 File — (ZIP) [file pone.0127317.s001.zip › ARLUNZ_Forestry_GhG_40_NetworksOff.png]

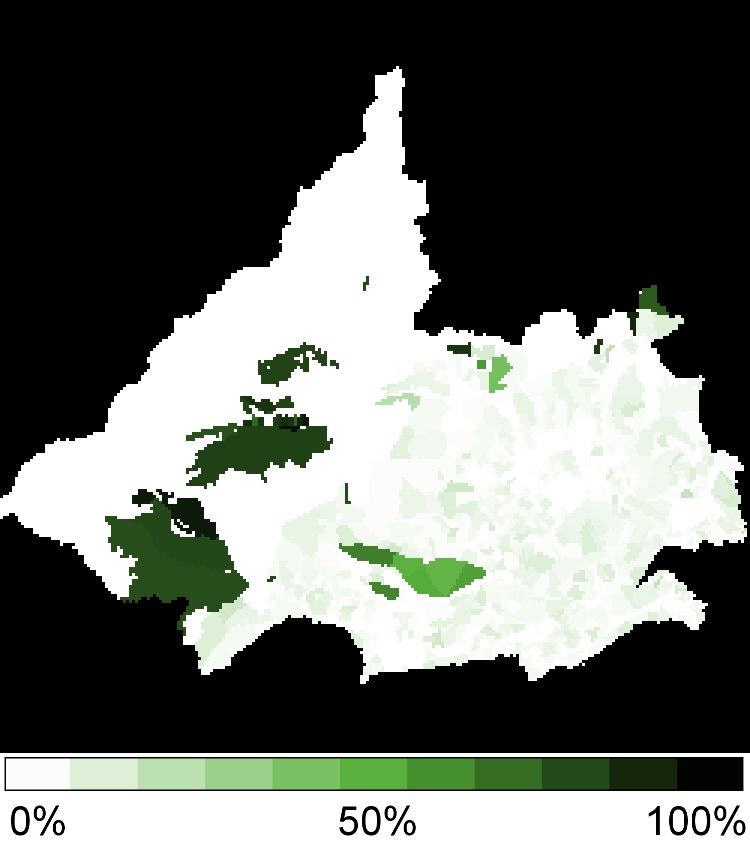

Supplement: S1 File — (ZIP) [file pone.0127317.s001.zip › ARLUNZ_Forestry_GhG_40_NetworksOn.png]

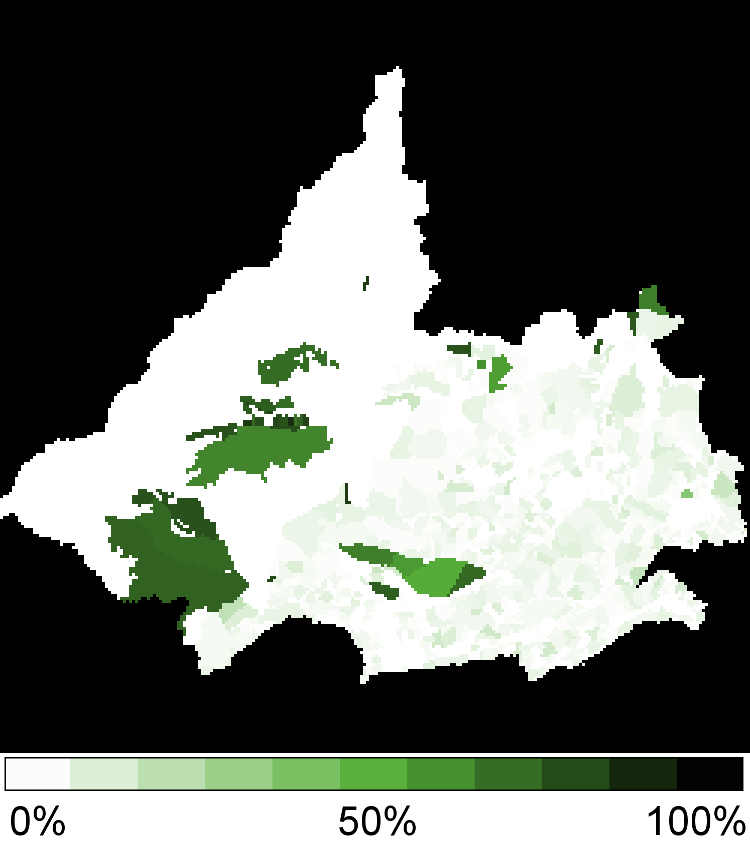

Supplement: S1 File — (ZIP) [file pone.0127317.s001.zip › ARLUNZ_Forestry_GhG_60_NetworksOff.png]

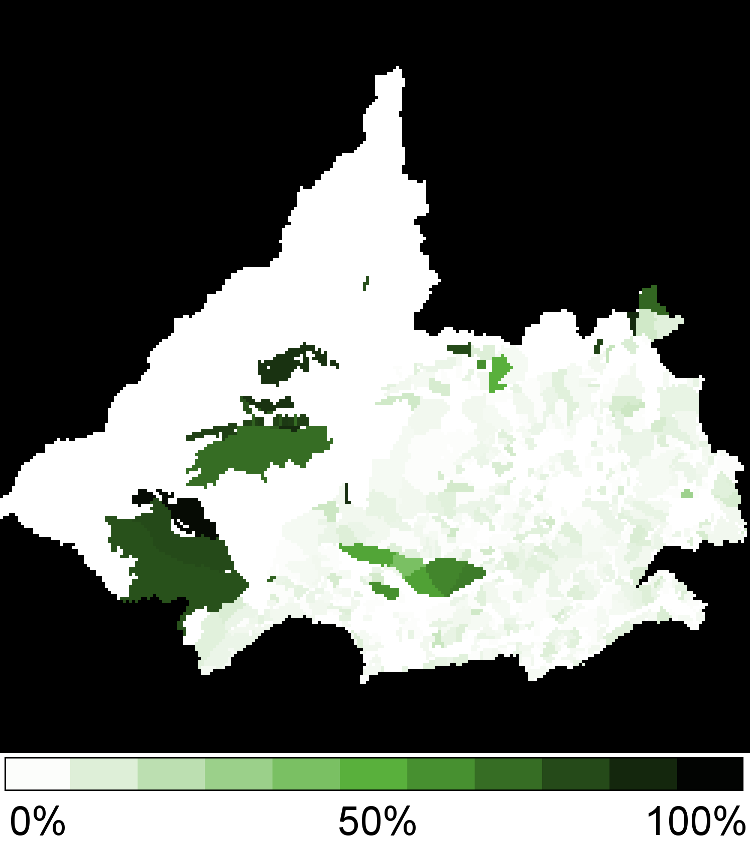

Supplement: S1 File — (ZIP) [file pone.0127317.s001.zip › ARLUNZ_Forestry_GhG_60_NetworksOn.png]

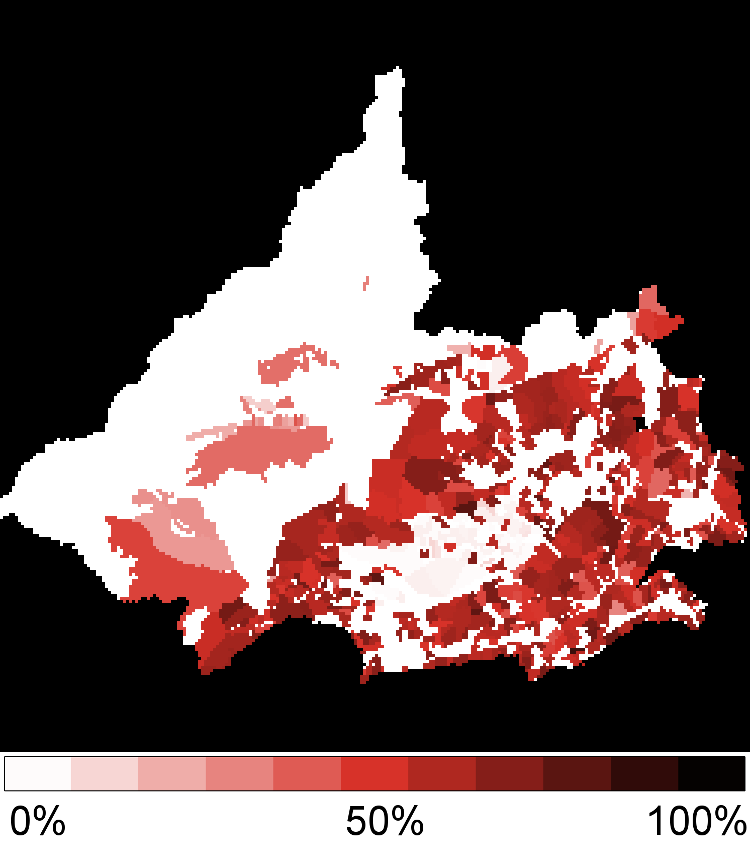

Supplement: S1 File — (ZIP) [file pone.0127317.s001.zip › ARLUNZ_SheepAndBeef_GhG_0_NetworksOff.png]

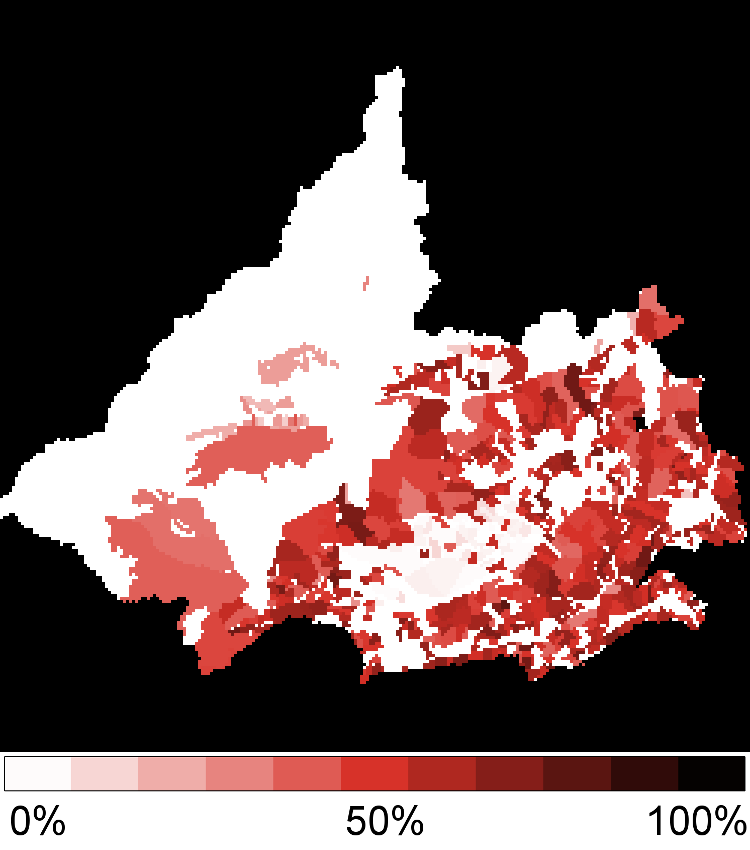

Supplement: S1 File — (ZIP) [file pone.0127317.s001.zip › ARLUNZ_SheepAndBeef_GhG_0_NetworksOn.png]

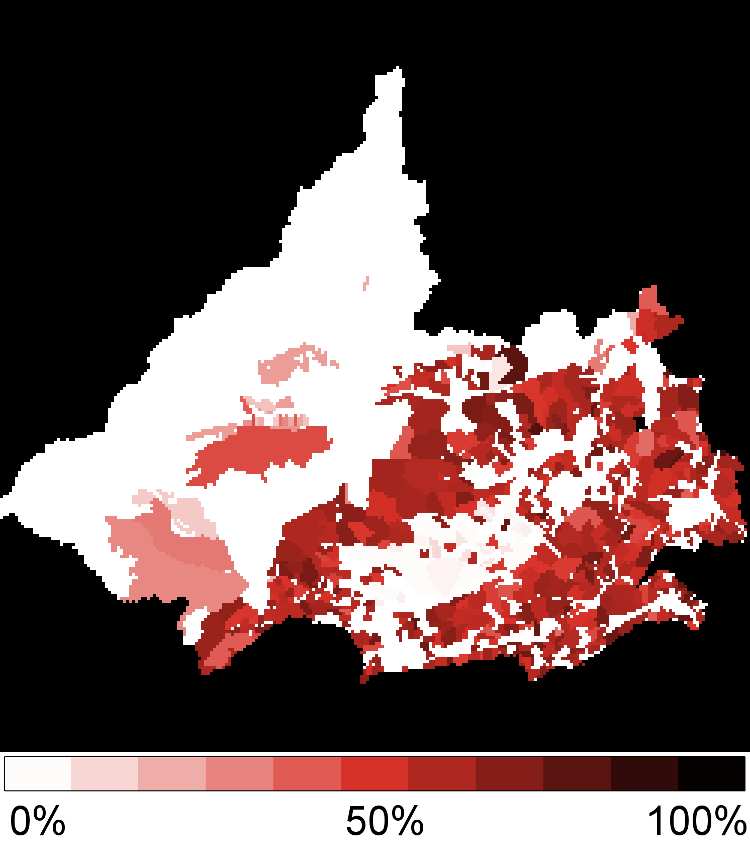

Supplement: S1 File — (ZIP) [file pone.0127317.s001.zip › ARLUNZ_SheepAndBeef_GhG_20_NetworksOff.png]

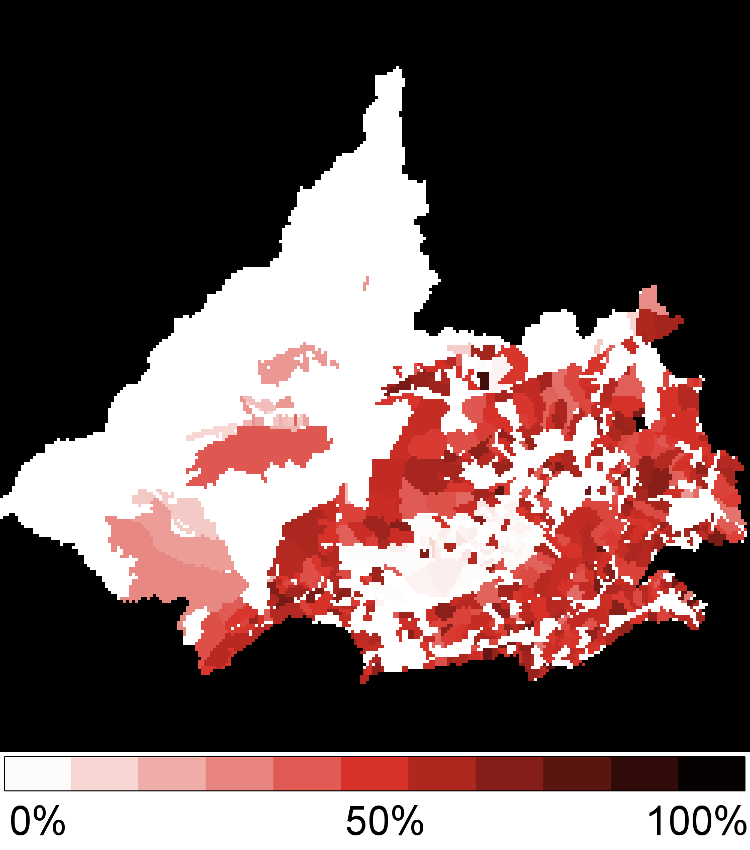

Supplement: S1 File — (ZIP) [file pone.0127317.s001.zip › ARLUNZ_SheepAndBeef_GhG_20_NetworksOn.png]

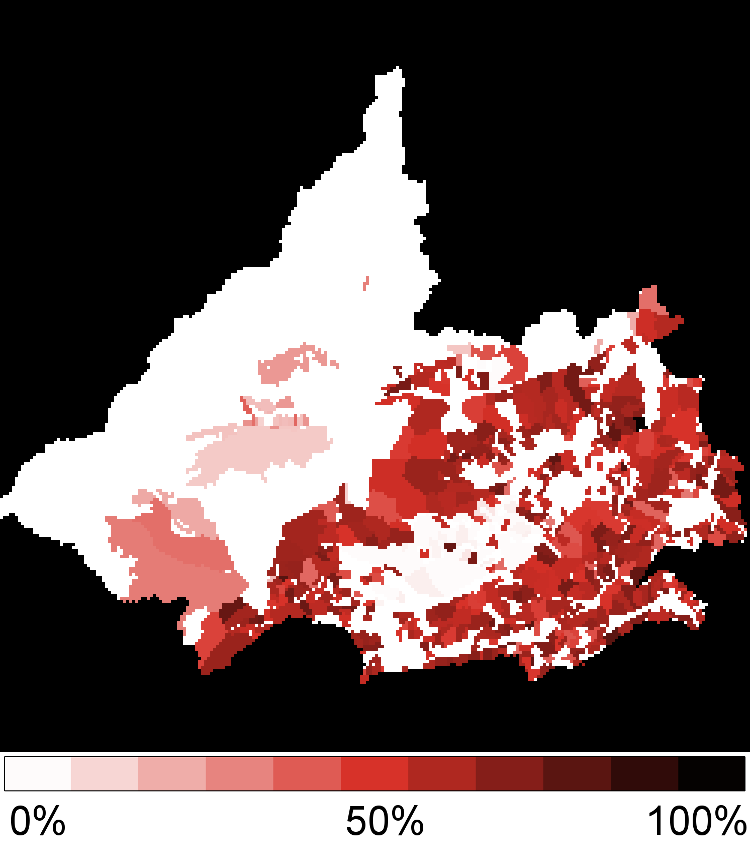

Supplement: S1 File — (ZIP) [file pone.0127317.s001.zip › ARLUNZ_SheepAndBeef_GhG_40_NetworksOff.png]

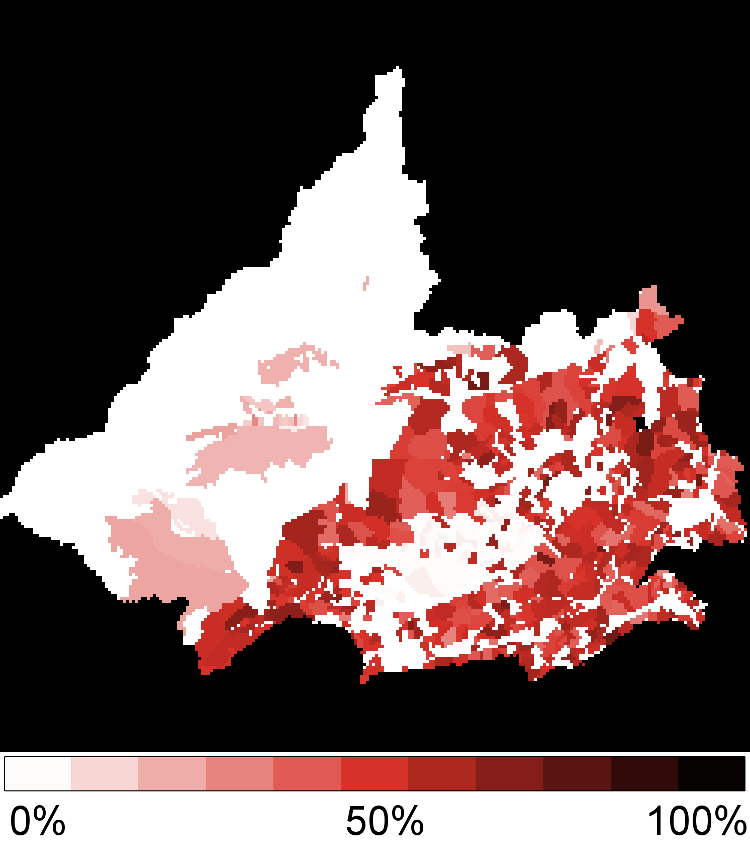

Supplement: S1 File — (ZIP) [file pone.0127317.s001.zip › ARLUNZ_SheepAndBeef_GhG_40_NetworksOn.png]

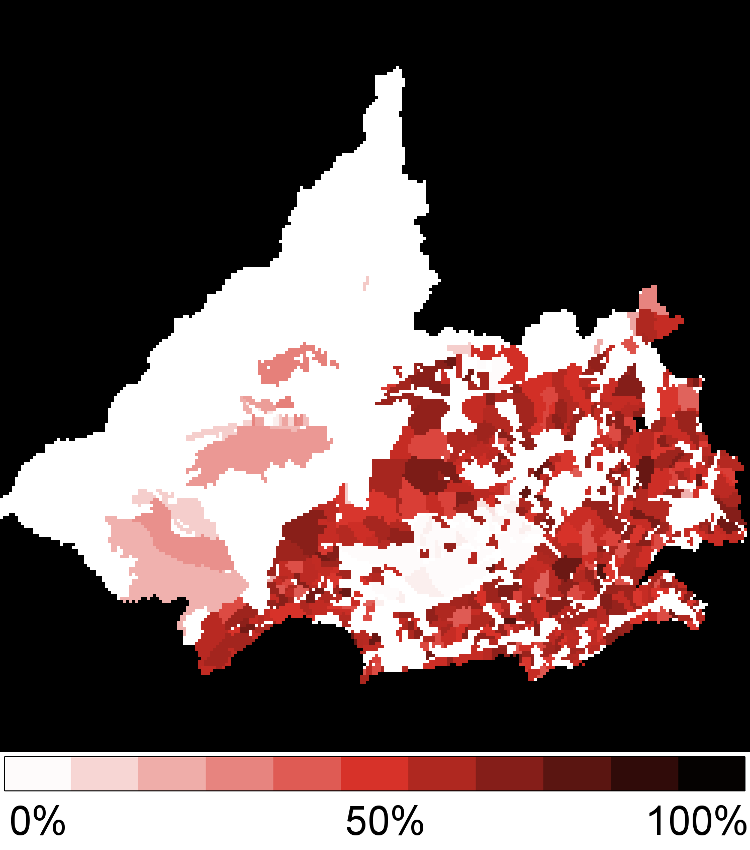

Supplement: S1 File — (ZIP) [file pone.0127317.s001.zip › ARLUNZ_SheepAndBeef_GhG_60_NetworksOff.png]

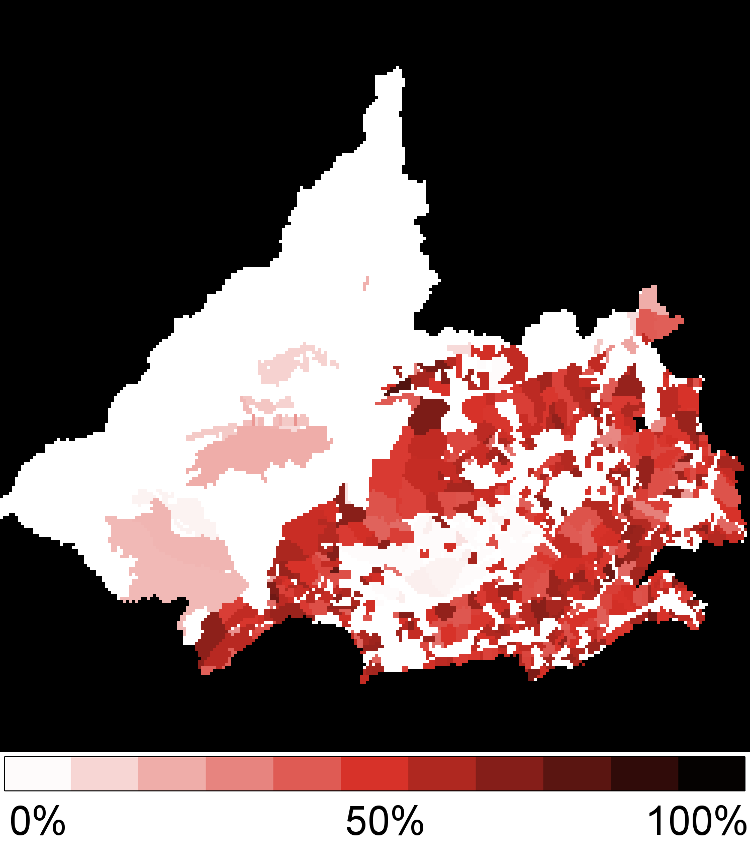

Supplement: S1 File — (ZIP) [file pone.0127317.s001.zip › ARLUNZ_SheepAndBeef_GhG_60_NetworksOn.png]
